# Nanoplatforms for Magnetic‐Photo‐Heating of Thermo‐Resistant Tumor Cells: Singular Synergic Therapeutic Effects at Mild Temperature

**DOI:** 10.1002/smll.202310522

**Published:** 2024-10-28

**Authors:** Binh T. Mai, Tamara Fernandez‐Cabada, John S. Conteh, Giulia E.P. Nucci, Sergio Fiorito, Helena Gavilán, Doriana Debellis, Lorenci Gjurgjaj, Teresa Pellegrino

**Affiliations:** ^1^ Italian Institute of Technology via Morego 30 Genoa 16163 Italy; ^2^ The Open University Affiliated Research Center Italian Institute of Technology via Morego 30 Genoa 16163 Italy; ^3^ Present address: School of Biological and Chemical Sciences University of Galway University Road Galway H91 TK33 Ireland; ^4^ Present address: Institut de Biotecnologia i de Biomedicina Universidad Autónoma de Barcelona (IBB‐UAB) Universitat Autònoma de Barcelona Edifici MRB, Carrer de la Vinya, s/n. Campus Cerdanyola del Vallès Barcelona 08193 Spain; ^5^ Present address: Departamento de Química Física Universidad Complutense de Madrid Madrid Spain

**Keywords:** combined cancer therapy, lysosomal permeabilization, mild hyperthermia, polymeric nanostructures, thermal resistant cancer

## Abstract

A self‐assemble amphiphilic diblock copolymer that can incorporate iron oxide nanocubes (IONCs) in chain‐like assemblies as heat mediators for magnetic hyperthermia (MHT) and tuneable amounts of IR780 dye as agent for photothermal therapy (PTT) is developed. MHT‐heating performance of photobeads in viscous media have the same heat performances in water at magnetic field conditions of clinical use. Thanks to IR780, the photobeads are activated by infrared laser light within the first biological window (808 nm) with a significant enhancement of photo‐stability of IR780 enabling the raise of the temperature at therapeutic values during multiple PTT cycles and showing unchanged optical features up to 8 days. Moreover, the photobeads fluorescent signal is preserved once internalized by glioblastoma multiforme (GBM) cells. Peculiarly, the photobeads are used as toxic agents to eradicate thermo‐resistant GBM cells at mild heat, as low as 41 °C, with MHT and PTT both of clinical use. Indeed, a high U87 GBM cell mortality percentage is obtained only with dual MHT/PTT while each single treatment dose not provide the same cytotoxic effects. Only for the combined treatment, the cell death mechanism is assigned to clear sign of apoptosis as observed by structural/morphological cell studies and enhanced lysosome permeability.

## Graphical abstract

Here, magnetic nanostructures made of chain‐like iron oxide nanocubes and photothermal dyes enwrapped in amphiphilic polymers, are developed. The obtained nanostructures are eradicating, in vitro, thermo‐resistant glioblastoma cells at temperature as low as 41 °C only when this therapeutic temperature is reached by a combination of MH and PTT of clinical conditions, thus offering new alternative approaches to treat thermo‐resistant cancer.

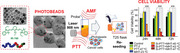

## 1 Introduction

Cancer is the second leading cause of mortality in Western World including Europe and North America.^[^ ^1^, ^2^ ^]^ The first line treatments include chemotherapy, radiotherapy and surgical resection and suffers a poor efficacy along with severe side effects.^[^ ^3^, ^4^ ^]^ As such, there is an urgent need to discover novel modality of treatment to tackle such disease. MHT is holding a tremendous potential.^[^ ^5^, ^6^ ^]^ In MHT, under an alternating magnetic field (AMF), magnetic nanoparticles (MNPs), administered at the tumor site, are used as antennas to convert the magneto energy into heat that can serve to kill cancer cells. The MHT toxic effect could occur due to a macroscopic temperature rise (41 – 46 °C) or due to intracellularly local disruptive heating effect without recording a perceptible temperature increase.^[^ ^5^, ^6^, ^7^, ^8^, ^9^, ^10^ ^]^ Indeed, MHT is in clinical trials to treat GBM and prostate cancer.^[^ ^11^, ^12^ ^]^ However, MHT alone suffers some drawbacks such as the difficulty to achieve a complete tumor reduction and at the same time the high dose of magnetic nanomaterials required intratumorally to induce therapeutic hyperthermia effects impairs the tumor imaging by magnetic resonance imaging (MRI) modality used to monitor brain tumor progression.^[^ ^11^, ^13^, ^14^, ^15^ ^]^ As such, several groups are developing magnetic nanoplatforms that can combine MHT and other therapies such as chemotherapy, radiotherapy, phototherapy to harness the performance of MHT in cancer and use less dose of magnetic materials.^[^ ^11^, ^16^, ^17^, ^18^, ^19^ ^]^

On the other hand, in PTT, light absorbing agents convert the energy of laser irradiation into heat to eradicate the cancer cells.^[^ ^20^, ^21^, ^22^ ^]^ In comparison to MHT, PTT achieves a hyperthermic condition at much lower dose of light absorbing agents. However, PTT can be applied only to superficial tissues. Near infrared (NIR) absorbing agents with absorption in the range 700 to 1100 nm, are targeted since the optical density of skin and tissue is minimal in this range.^[^ ^20^, ^21^, ^22^ ^]^ Although, the tissue light scattering and penetration is a major concern of PTT,^[^ ^21^ ^]^ the combination with MHT heating represents an interesting combined approach to achieve a therapeutic temperature without the risk of using harsh conditions of laser power and of AMF field conditions. In this context, the development of nanomaterials that can combine MHT and PTT could provide novel cell death mechanisms leading to synergistic therapeutic stress effects for cancer treatment that apparently are not manifest when MHT and PTT are applied separately. As such, the synthesis of nanostructures that respond to MHT and PTT has been an active and fast‐growing field during the last few years thanks to their great potential in cancer therapy.^[^ ^23^, ^24^, ^25^ ^]^

To integrate the NIR absorbing moieties that can generate heat upon laser exposure, plasmonic metals (Ag and Au) and copper chalcogenides (Cu_2‐x_S) nanoparticles at different shapes such as nanorods, nanoshells and nanospikes have been used. However, these materials always pose concern about their potential toxicity and their accumulation in the body.^[^ ^23^, ^26^, ^27^, ^28^, ^29^, ^30^, ^31^, ^32^ ^]^ Alternatively, photothermal agents that are based on organic dyes such as heptamethine dyes, which possess sharp and distinct absorption peak thus providing an outstanding PTT conversion efficiency, are often considered.^[^ ^33^ ^]^ Among the heptamethine NIR dyes, IR780 has high optical extinction coefficient in the range from 700 to 1000 nm, it is cost effective, and displays the highest photothermal conversion efficiency among other dyes class.^[^ ^33^, ^34^, ^35^ ^]^ On the other hand, for MHT, cubic‐shaped iron oxide MNPs not only have good biocompatibility and biodegradability,^[^ ^36^, ^37^, ^38^ ^]^ but also, thanks to their shape, exhibit magnetic heat losses under AMF, that are much higher than classical spherical iron oxide MNPs under clinical MHT conditions. Indeed, nanocubeof iron oxide at a comparable magnetic volume of spherical IONPs possess specific absorption rate (SAR) values that are one order magnitude higher than the spherical nanoparticles of similar composition.^[^ ^39^, ^40^, ^41^ ^]^ Moreover, IONCs themselves when composed of Fe_3_O_4_ were demonstrated to be able to amplify the heating efficiency when exposed to bimodal treatment of MHT and PTT.^[^ ^41^, ^42^, ^43^, ^44^ ^]^

Aiming at providing novel nanoplatforms that can respond to combined MHT and PTT in a more clinical‐relevant conditions for cancer therapy and for heat‐resistant GBM cells in particular, we have developed self‐assembled nanostructures of an amphiphilic polymer, IONCs and IR780. Taking advantage of photo‐induced atom transfer radical polymerization and Diels‐Alder chemistry, we designed and synthesized an amphiphilic diblock copolymer named PEG‐*b*‐P(DiolMAm‐co‐BenzylMAm) that has PEG segment for water stability while the other block contains propandiol and benzyl pendant, for assigning anchoring and hydrophobic features to the polymer. Indeed, the propandiol side groups offers a good affinity between IONCs and polymer chain and, therefore, provide a higher compatibility to the P(DiolMAm‐*co*‐BenzylMAm) domain anchored on IONCs. Instead, the benzyl pendants offer a high affinity between the polymer and the IR780 dye or with the IONCs surface via p‐p stacking interactions enabling the self‐assembling and the encapsulation process. Here, we found that IONCs and IR780 could be enwrapped within the PEG‐*b*‐P(DiolMAm‐*co*‐BenzylMAm) polymer by means of a self‐assemble process. In the nanobeads, the IONCs tend to spontaneously assemble in short chains while the amount of loaded IR780 is tunable and has a great impact on the morphologies of resulting nanostructures (photobeads). The SAR values of photobeads measured using AMF conditions respecting the biological limit (*H*x*f* < 5.10^9^ A/m·s) are high and comparable to those of standard PEGylated IONCs while dropped by only 10% to 20% in highly viscous media (up to ≈97.3 mPa s). Notably, a sustained increase of the temperature of photobeads solution was recorded using a mild condition of laser at 808 nm (< 1.1 W cm^−2^) while the increase of temperature can be boosted by applying laser and MHT in parallel. Most importantly, when applying MHT and PTT on our photobeads administered to U87 GBM cells in vitro, we found that only the combined dual treatment applied either subsequently or simultaneously, even at temperature as low as 40 °C, enabled to reach a very high mortality (≈90%) of thermal resistant U87 cells and instead they resisted at temperature as high as 47 °C when immersed in thermal bath, as here demonstrated. The same efficacy was also achieved when the treatment was extended to epidermoid carcinoma cells (A431). A pronounced apoptosis and lysosome activity were recorded only in case of cells that were treated with combined MHT and PTT with respect to each individual treatment. Based on our results, we indicate that the mechanism by which the combined MHT and PTT treatment on our photobeads were toxic even at mild temperature, is related to the permeabilization of lysosomes compartment thus provoking cell death and acidification of the cytosol while triggering cytotoxicity.

## 2 Results and Discussion

### 2.1 Amphiphilic Polymer Synthesis and Characterization

**Figure 1:**
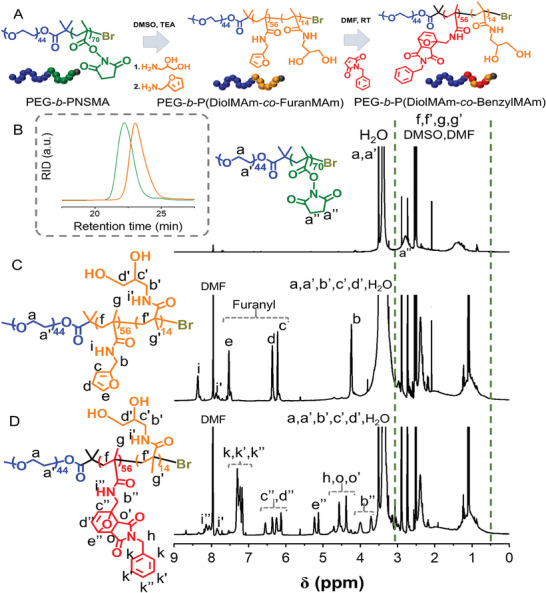
Summary of the amphiphilic diblock copolymers synthesis and NMR characterization. a) Scheme of the synthetic approach used to produce diblock copolymer made of PEG, benzyl pendant and diol derivate. Starting with the synthesis of a diblock copolymer made of PEG and activated ester *N*‐succinimidyl methacrylate (NSMA) gives PEG‐*b*‐PNSMA polymer. The NSMA derivate is used as reactive precursor in the second step to introduce propandiol and furfuryl side groups by means of aminolysis reaction. The latter was then clicked with benzyl maleimide in a last reaction step to yield a hydrophobic polymer that has Diels‐Alder adducts as side groups. b) ^1^H NMR spectrum of PEG‐*b*‐PNSMA recorded in DMSO‐d_6_, inset: the SEC traces of pristine PEG‐*b*‐PNSMA (green) and the one upon the aminolysis reaction with furfuryl amine and 3‐amino‐1,2‐propanediol. c and d) depict the ^1^H NMR spectra of PEG‐*b*‐P(FuranMAm‐*co*‐DiolMAm) and the one after the Diels‐Alder reaction with benzylmaleimide (PEG‐*b*‐P(BenzylMAm‐*co*‐DiolMAm), respectively.

To co‐encapsulate IONCs and IR780 within a single polymeric structure, we developed a tailor‐made amphiphilic diblock copolymer having side groups of benzyl and diols as illustrated in scheme of **Figure** **1**A.

In the first step, Atom Transfer Radical Polymerization (ATRP) Bromide initiator functionalized with PEG (PEO‐Br) was used as macro‐initiator to grow a second block of activated ester *N*‐succinimidyl methacrylate (NSMA) by means of photo‐ATRP technique.^[^ ^45^, ^46^, ^47^ ^]^ The successful formation PEG‐*b*‐PNSMA was confirmed by ^1^H NMR as shown in Figure 1B in which the characteristic resonant signal of four protons of maleimide group (a″, δ 2.8 ppm) could be clearly detected. By comparing the integration between protons of NSMA (a″) and the ones of PEG segment (a, a′), a degree of polymerization of 70 was roughly estimated for PNSMA block. Size Exclusion Chromatography (SEC) revealed that PEG‐*b*‐PNSMA has a molar mass of 14 500 g mol^−1^ along with a dispersity (Đ) as low as 1.2 (Figure 1B, inset) which indicated that the photo‐ATRP of NSMA occurred in a controlled manner. The PEG‐*b*‐PNSMA was then reacted to a mixture of furfuryl amine (FMA) and 3‐amino‐1,2‐propanediol (APD) precursors to yield PEG‐*b*‐P(FuranMAm‐*co*‐DiolMAm) via the aminolysis reaction between NSMA and primary amines. Here, we fixed the ADP/FA molar ratio of 1 to 4 to ensure that the resulting PEG‐*b*‐P(FuranMAm‐*co*‐DiolMAm) is sufficiently water soluble. In Figure 1C, the ^1^H NMR spectrum of PEG‐*b*‐P(FuranMAm‐*co*‐DiolMAm) shows the aromatic characteristic peaks of furanyl ring (protons c, d, e of Figure 1C). Instead, the appearance of amide proton i' in Figure 1C indicated the existence of propanediol group in resulting copolymers. The difference of integration between proton i and i' revealed a ratio between furfuryl methacrylamide and propanediol methacrylamide of 82:18% which is in good agreement with the feeding ratio (80/20). The polymer upon the aminolysis reactions features a reduced molar mass of 12 500 g mol^−1^ while a low PDI of 1.21 remained unchanged (Figure 1B, inset). As expected, the Dynamic Light Scattering (DLS) shows that the d_H_ of PEG‐*b*‐P(FuranMAm‐*co*‐DiolMAm) dissolved in water is 15 and 10 nm if weighted by intensity and number, respectively (Figure S1A, Supporting Information), thus indicating that PEG‐*b*‐P(FuranMAm‐*co*‐DiolMAm) is fully soluble in water. To render the P(FuranMAm‐*co*‐DiolMAm) polymer amphiphilic, the hydrophobic benzyl maleimide precursor was clicked to the furfuryl side groups of the polymer chain by means of Diels‐Alder chemistry to finally yield PEG‐*b*‐P(DiolMAm‐*co*‐BenzylMAm). A quantitative conversion of furfuryl group to Diels‐Alder adducts was verified by ^1^H NMR (Figure 1D). Here, the signals of furanyl ring are completely retracted while the ones of benzyl ring (proton k, k′ and k″ in Figure 1D) and Diels‐Alder adducts (c″, d″, e″, o, o′ in Figure 1D) can be clearly visualized. The insertion of benzyl group as side chain is aimed to assist the encapsulation of IR780 and hydrophobic‐capped IONCs via π–π stacking interaction. The PEG‐*b*‐P(DiolMAm‐*co*‐BenzylMAm) polymer alone, self‐assembled in aqueous solution (by dissolved polymer powder in DMF as non‐selective solvent and followed by the addition of water as selective solvent, see detailed protocol in the experimental section) to form a nanostructure having hydrodynamic diameter (d_H_) of 160 nm (weighted by number) which might be assigned to polymersome structure (Figure S1B, Supporting Information).

### 2.2 Photobeads Preparation and Their Structural Characterization

PEG‐*b*‐P(DiolMAm‐*co*‐BenzylMAm) was then used as main polymer to encapsulate IONCs and IR780, forming photobeads. IONCs (14 nm) were prepared accordingly to a solvothermal process developed by us and chosen because of their optimal magneto energy losses and their superparamagnetic behavior which being less interactive facilitate the encapsulation step.^[^ ^39^, ^48^ ^]^ PEG‐*b*‐P(DiolMAm‐*co*‐BenzylMAm) and IR780 were found to be soluble in a mixture of 25:75 part in volume of THF/DMF solution while as synthesized IONCs, capped with oleic acid, precipitated out of this solution. Therefore, to render IONCs soluble in such media, the oleic acid surfactant at the surface of IONCs were exchanged with DOPA‐BAmBr (Figure S2A, Supporting Information).^[^ ^42^ ^]^ Upon the ligand exchange, the DOPA‐BAmBr capped IONCs were found to be well soluble in a mixture of THF/DMF (25/75) forming a clear brown solution. DLS measurements of IONCs‐DOPA‐BAmBr measured in such media reveals a low size, with d_H_ of 14, 18, and 23 nm weighted by number, volume and intensity, respectively (Figure S2B, Supporting Information), indicating the fully dispersion of the surface‐modified IONCs in their individual state.

**Figure 2:**
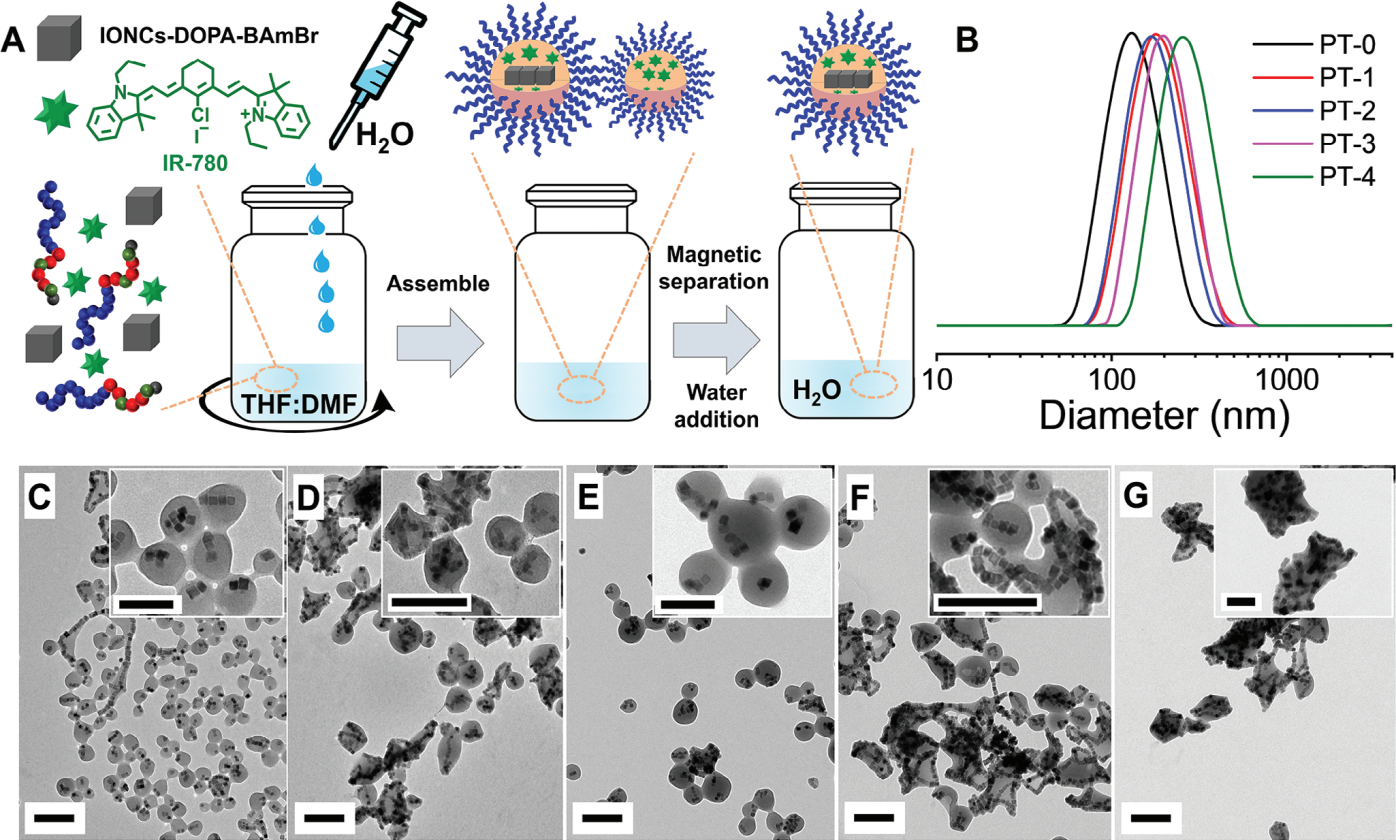
Preparation of polymer nanobeads encapsulating IONCs and IR780 via the self‐assembly process. a) Schematic illustration of the self‐assembly process to prepare magnetic photobeads. The first step involves the addition of a selection solvent (H_2_O) into a solution of surface modified IONCs, polymer and IR780 dissolved in a non‐selective solvent mixture (THF/DMF). Magnetic photobeads were cleaned by means of magnetic separation. DLS traces b) (weighted by number) and TEM images c–g) of obtained magnetic photobeads when different amounts of IR780 were used. C, D, E, F and G correspond to samples PT‐0, PT‐1, PT‐2, PT‐3, PT‐4 (TEM scale bar of 200 nm for low magnification and 100 nm for the inset figures at higher magnifications). In PT‐0, no IR780 was added while the feeding dye to Fe ratios (µg per mg) was 125, 188, 219 and 313 for PT‐1, PT‐2, PT‐3 and PT‐4 respectively.

Next, to obtain the magnetic‐Photobeads a self‐assembly process was followed (**Figure** **2**A). In the first step, the polymer, the due amounts of IR780 and the functionalized nanocubes (IONC‐DOPA‐BAmBr) were dissolved in the THF/DMF (25/75% in volume) mixture to form a homogeneous solution. Next, the formation of photobeads was induced upon addition of water at a rather quick rate (8 mL min^−1^). The solution turned from clear dark brown to milky brown soon after water addition as a first indication of beads formation. Free polymeric beads which had no magnetic IONCs were easily removed by means of magnetic collection of the magnetic‐photobeads as a pellet to a commercial magnet (0.3 T) and discarding the supernatant solution. The pellet was then redissolved in fresh water. Here, we noticed that the incorporation of IR780 influenced the d_H_ and morphologies of the resulting photobeads (Table S1, Supporting Information). The photobeads without IR780 (PT‐0) had a d_H_ (weighted by intensity and reported with the half width at half maximum, HWHM) of 140 ± 100 nm while by increasing the amount of IR780 (PT‐1, ‐2, ‐3, ‐4) made the size of magnetic‐Photobeads gradually bigger (Figure 2B). The d_H_ weighted by number and volume (Table S1, Supporting Information) also revealed an identical trend. Interestingly, IONCs within the beads had the trend to assemble within compact polymer beads in form of short chains, which were observed with and without the addition of IR780 (Figure 2C–G).

Indeed, the tendency of IONCs to form short chains still remained once small amounts of IR780 were introduced (less than 125 µg mgFe^−1^ for PT‐1 and less than 188 µg mgFe^−1^ for PT‐2, Figure 2D and E). The addition of larger amount of IR780 (≈219 µg mgFe^−1^) led to the formation of magnetic‐Photobeads in which IONCs formed quite long chain (PT‐3, Figure 2F) while an even further increase of IR780 amount (313 µg mgFe^−1^) resulted in magnetic‐Photobeads with much less well‐defined nanostructures as interconnected chains (PT‐4, Figure 2G). This may be explained by the fact that phenyl ring and their substituent have been often involved in the assemblies of nanoparticles in chains as previously reported.^[^ ^49^, ^50^ ^]^ On the other hand, this specific arrangement of IONCs is of particular interest for MHT applications as it has been reported that the chain‐like configuration of magnetic nanoparticles under AMF promote magnetic dipole interactions which improve the heating capacity of magnetic nanostructure.^[^ ^50^, ^51^, ^52^, ^53^, ^54^ ^]^

The successful encapsulation of IR780 within the photobeads was verified by absorption spectroscopy. The absorption spectra of the four magnetic photobeads samples showed the characteristic absorption peak of IR780 in the region 780 – 810 nm which has a similar profile to that of the free dye in DMF (Figure S4A and B, Supporting Information). It was not trivial to record the spectrum of IR780 in water as this dye is not water soluble and undergoes rapid decomposition.

### 2.3 Optical, Magnetic Hyperthermal and Photothermal Characterizations

Interestingly, the encapsulation of the IR780 dye within polymer beads induces a peak shift in maximal of the dye from 780 to 810 nm (Figure S4A, Supporting Information) which matches well with the wavelength of laser used in PTT (808 nm). This red shift could be attributed to the π–π interaction between two dye molecules and/or between the dye and phenyl groups of the hydrophobic side‐domain of the polymer.^[^ ^34^, ^55^ ^]^ As the optical density at 808 nm increases, an improvement of photo‐thermal efficacy might be anticipated with respect to the dye alone in solution. To determine the amount of loaded IR780 for the photobeads prepared at gradually increased amounts of dye, the photobeads were dissolved in a mixture of 10% in volume of H_2_O in DMF. In this media, the polymer is soluble and thus making photobeads disintegrate. IONCs were settled down by centrifugation and the supernatant was collected and subjected to NIR absorption measurement. The concentration of IR780 was quantified using a calibration curve of IR780 in H_2_O/DMF (10:90 in volume). As the dye absorbs in NIR region, there is no interference between the spectrum of released dye and the one of polymer. As shown in **Table** **1**, the amount of loaded IR780 can be tuned by changing the feeding amount of IR780.

**Table 1:** The dependence between the loaded and the feeding amounts of IR780.

Sample	IR780/Fe feeding [mg/mg]	IR780/Fe encapsulated [mg/mg]	% of trapped IR780
PT‐1	125	25	20
PT‐2	188	72	38
PT‐3	219	38	18
PT‐4	313	65	21

**Figure 3:**
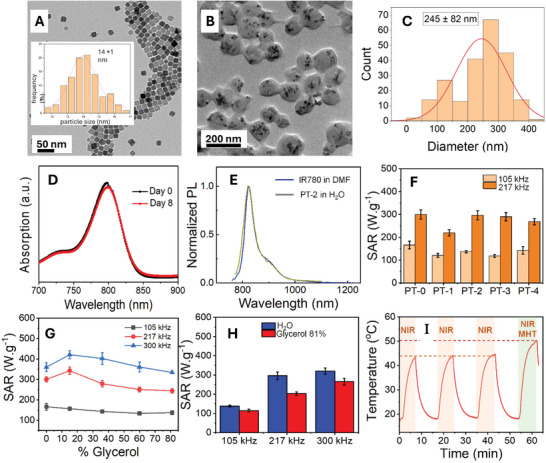
Magnetic hyperthermia and photothermal properties characterizations of photobeads. A) TEM image of IONCs with inset the histogram of their TEM size distribution. B) TEM images and C) Histogram of TEM size distribution of PT‐2 Photobeads, respectively. D) Vis‐NIR absorption spectra of PT‐2 photobeads samples having optimal loading capacity of IR780 at soon after the preparation (day 0, black dots) and at day 8 (red dots). E) Normalized Photolµminescent (PL) spectra of IR780 dye in DMF and photobeads that contains 7.2 µg of IR780 per mgFe (PT‐2). To note, all data points of the PL spectra of each of the samples were divided by the maximal emission intensity recorded at 810 nm to make the maximal emission in PL spectra equals to 1. F) SAR values of photobeads containing different amounts of IR780. G) SAR values of IR780 free photobeads (PT‐0) as a function of glycerol concentration. H) The comparison between SAR values of PT‐2 measured in viscous media (81% glycerol) and in water at 24 kA m^−1^ at well‐defined frequencies (105, 217, and 300 kHz) and I) The heating profiles of PT‐2 upon the exposure to laser of 808 nm (4.67 W cm^2^) (first three cycles shaded in red) and combined MHT (24 kA m^−1^, 120 kHz) and PTT (last cycle shaded in green).

Among the four samples prepared, the optimal conditions to achieve the highest entrapping dye percentage was identified for PT‐2 in which the highest percentage of dye encapsulation corresponding to 38% of the feeding IR780 was loaded within photobeads along with a highest amount of IR780 per mg Fe (72 µg of dye/1 mg Fe of magnetic‐Photobeads). Along with the highest dye encapsulation amount, PT‐2 sample was also characterized by a fair degree of homogeneous size distribution resulting in photobeads of ≈245 ± 82 nm as analyzed by TEM and each bead incorporates multiple nanocubes with cube edge of ≈14 ± 1 nm (**Figure** **3**A–C). Moreover, the maximal optical density of PT‐2 at 800 nm, stored in dark at ambient condition, decreased by less than 5% even after a prolonged storage up to 8 days (Figure 3D).

We also noticed that the encapsulation within polymer matrix significantly enhanced the optical stability of IR780 dye in comparison with other studies reported in literature where lipidic or other polymeric nanostructures were used.^[^ ^56^, ^57^, ^58^ ^]^ Indeed, other IR780 dye loaded polymer micelles or lipid nanoparticles showed a drop of optical density at 800 nm by 50% within a day.^[^ ^56^, ^57^, ^58^ ^]^ This better stability of our system could be explained by a compact and rigid hydrophobic domain made of aromatic moieties of the polymer and alkyl surfactants at the nanocubes surface that prevent the contact between water and loaded dyes and thus suppressing their rapid degradation. Indeed, Figure 3E represents the photoluminescent (PL) spectra of IR780 dye in DMF and the one encapsulated in PT‐2 in water. Here, an identical emitting feature was obtained. The comparison between PL of IR780 loaded in magnetic‐photobeads and the one in water was not possible as this dye is not water soluble and decomposed quickly in contact with water. To further study the stability of photobeads in physiological‐relevant conditions, we monitored the evolution of photobeads hydrodynamic size, d_H,_ in saline (0.9% NaCl) and Phosphate‐buffered saline (PBS, at pH ≈ 7.4) as function of storing time. Interestingly, PT‐2 photobeads were very stable in such media. While in saline solution the peaks recorded at different days over imposed, in PBS, the peaks maxima shifted at lower hydrodynamic size from day zero to day 1 but then shows marginal changes in the d_H_ profile up to 8 days of storage (Figure S3, Supporting Information).

We then evaluated the heating ability of the magnetic photobeads by calorimetric measurements upon exposure to AMF at values of field intensity (24 kA m^−1^) and frequencies (105 or 217 kHz), which both respect the biological limit. All the magnetic photobeads showed very high SAR values at the investigated AMF conditions (Figure 3F). The IR780 free photobeads (PT‐0) has SAR values (160 and 290 W g^−1^ at 105 and 217 kHz, respectively) very similar to that of photobeads incorporating also the IR780 dye. It is important to note that these SAR values are comparable to the ones of single PEGylated IONC having the same edge size reported in our previous study (130 W g^−1^ at 24 kA m^−1^ and 100 kHz).^[^ ^59^ ^]^ These data suggest that incorporation of IR780 into the magnetic‐Photobeads, which induces a notable increase of d_H_, does not significantly affect the SAR values of resulting photobeads. This is because such tiny nanocubes (14 nm) have a Neel‐based heating mechanism and if the aggregation of multiple cubes in the beads may induce a reduction in SAR, the chain like assembly may still compensate the loss.^[^ ^60^ ^]^ We have also studied by AC magnetometer the magnetic heat losses in viscous media that simulate the viscosity in tumor or intracellular sub‐compartments, which are often critical conditions for many magnetic materials designed for MHT.^[^ ^9^, ^61^, ^62^, ^63^, ^64^, ^65^ ^]^ This study was first conducted with PT‐0 sample, the magnetic photobeads loaded with only magnetic nanocubes and no dye. PT‐0 sample was dissolved in a mixture of water–glycerol at increased volume amount of glycerol (from 15% to 81%). Interestingly, the SAR values of PT‐0 did not depend on the media viscosity at all the frequencies studied (Figure 3G). At 105 kHz, the increase of solution viscosity to 81% resulted in only a slight drop (≈10%) of SAR values. The same observations were made with PT‐0 sample when the measurements were performed at 217 and 300 kHz. Next, the IR780 loaded magnetic‐Photobeads (PT‐2 sample) was also tested for SAR measurement in solution of 81% glycerol thus at the highest viscosity. Having a similar morphology to PT‐0, the heating performance of PT‐2 were also found to be independent on solution viscosity and only a marginal decrease (≈10%) of SAR measured at 105 kHz was recorded (Figure 3H). When higher frequencies of 217 and 300 kHz were applied, higher drops of 25% and 15% were recorded, respectively. The almost‐independent viscous magnetic heating could make these nanoplatform appealing for intratumoral or intracellular heating.

To test the response of the magnetic photobeads to laser and AMF excitation, in this proof of concept experiment, a solution of PT‐2 ([Fe] = 2.6 g L^−1^) was exposed to three cycles (of 7 min each) of 808 nm laser irradiation (power density of 4.67 W cm^2^). This was directly followed by a fourth and last cycle performed under MHT and PTT applied simultaneously (using the same laser conditions and an AMF of 24 kA m^−1^ and 120 kHz (the set up used for the combined MHT+PTT is shown in Figure S5, Supporting Information). An increase of temperature difference of 26 °C was recorded during the first cycle of PTT treatment (Figure 3I). Moreover, the same heating profile was maintained upon multiple PTT treatments of PT‐2 applied one after the other for a total of three cycles.

The observed outstanding PTT performance could be due to an improvement in terms of stability of encapsulated IR780 as described above. Next, on the same sample, when the AMF and laser were applied simultaneously, a final temperature of 50 °C could be reached, which corresponded to an overall increase of 6 °C higher with respect to only PTT. We noted, however, that polymeric beads in which only IR‐780 dye was incorporated (without IONCs) when subjected to a 808 nm laser exposure at 4.67 W cm^2^ for 3 cycles of 7 min and each followed by 5 min of laser off, the heating performance of such photobeads deteriorated at the second cycle of PTT (Figure S9, Supporting Information) with a photothermal conversion efficiency of ≈36%. This observation highlights the importance of the co‐encapsulation of IONCs to stabilize the PTT performance of photobeads.

It is generally assumed that the PTT induces the degradation of the photothermal dyes and, therefore, the exploitation of nanostructures containing such organic molecules is not feasible for multiple treatments.^[^ ^33^ ^]^ This could be related to the presence of nanocubes, which absorb part of the light, thus enabling a protection of the dye by photo‐degradation. Given the presence of Diels‐Alder adducts in the polymer structure, a thermal degradation of the polymer in response to increase of temperature during the combined MHT+PTT treatment may be expected. However, as a first observation, the heat performance of photobeads during multiple cycles of PTT remained unchanged consistent with the hypothesis of the maintenance of the photobeads structure. Despite the colloidal stability of the magnetic photobeads as measured by their hydrodynamic size, during heat treatment (i.e., combined MHT and PTT), we observed a small but significant difference between the absorption spectra of the supernatant of the MHT and PTT dual treated sample with respect to that of non‐treated sample (Figure S7, Supporting Information). Please note that the absorption spectra were measured on the supernatant upon magnetic collection of the magnetic photobeads to a 0.3T magnet. The absorption difference indicates that a small amount of the loaded dye was leaking out of the photobeads during the heat treatment. Next, we further performed the DLS measurement by exposing a polymer solution to a gradual temperature increase in the range from 25 to 50 °C, that is the maximum temperature reached during MHT+PTT dual treatment. Interestingly, no significant changes in hydrodynamic size were recorded by DLS, as the size recorded was always between 220 and 200 nm, indicating the high stability of the polymer during the combined treatment (Figure S6, Supporting Information).

This first part of the study was conducted at a power density of 4.67 W cm^2^ which is higher than the condition accepted in clinic. However, this condition was chosen in the proof‐of‐concept to demonstrate the capability of photobeads to respond to combined MHT and PTT treatment and, at the same time, to test stability of the materials in a more extreme condition. However, for the in vitro experiment with cells, a power density of ≈1.0 W cm^2^ was chosen for this study. This value is certainly lower than the biosafety limit and still, as shown in the next section, enabled us to demonstrate the suitable toxic effects of photobeads even under these clinical acceptable conditions.

### 2.4 NIR Bioimaging of Cells using Photobeads

**Figure 4:**
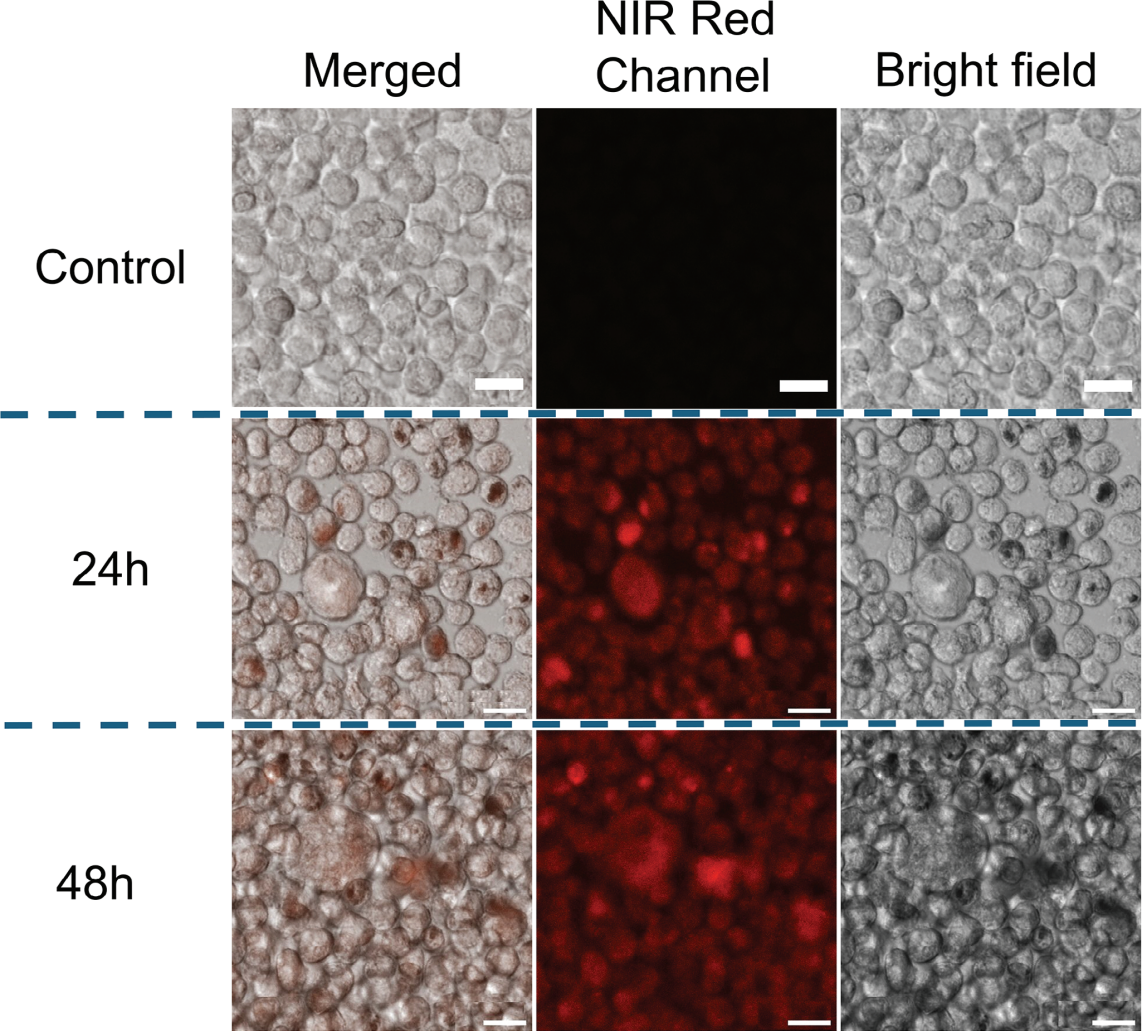
Confocal imaging of U87 GBM cells labelled with PT‐2 after 24 and 48 h of incubation and comparison to untreated U87 cells (control). NIR Red channel with excitation at 750 nm and collection of emission at 745 ± 35 nm, show a clear red PL signal indicating the internalization of the photobeads in U87 MG cells after 24 and 48 h of continuous incubation. Black spots in transmission light images correspond to the dark contrast of the magnetic photobeads. From these same images no sign of cell sufferance can be seen. Scale bar: 20 µm.

Featuring a high amount of loaded IR780 dye on PT‐2, we first investigated its potential as imaging probe in vitro on tumor cells and later as heat mediators for combined MHT and PTT. To check PT‐2 as NIR fluorescent marker, U87 GBM cells were cultured in 3D and incubated with PT‐2 magnetic photobeads for 24 or 48 h. After replacing the media to eliminate unbound and non‐internalized photobeads and split the spheroids in single cells using accutase to facilitate their imaging, confocal imaging analysis was performed at an excitation wavelength of 750 nm. A bright red signal appeared in the far‐red channel (745 ± 35 nm) on the U87cells treated with PT‐2 for 24 and 48 h, suggesting an efficient internalization by U87 cells (**Figure** **4**). Moreover, U87 cell seemed not to be affected by the magnetic‐photobeads at the dose used (3.18 gFe L^−1^) as the cells preserved their usual cell morphology. It is important to mention that in the transmission channel, dark spots on the cells were observed at 24 and 48 h (Figure 4). These spots that colocalized with the bright fluorescent spots in the merged channel are attributed to the contrast of PT‐2 beads, which may either adhere to the cell surface or be taken up intracellularly, as later shown by TEM characterization.

### 2.5 in vitro Therapeutic Efficacy of Photobeads in Combined MHT and PTT

**Figure 5:**
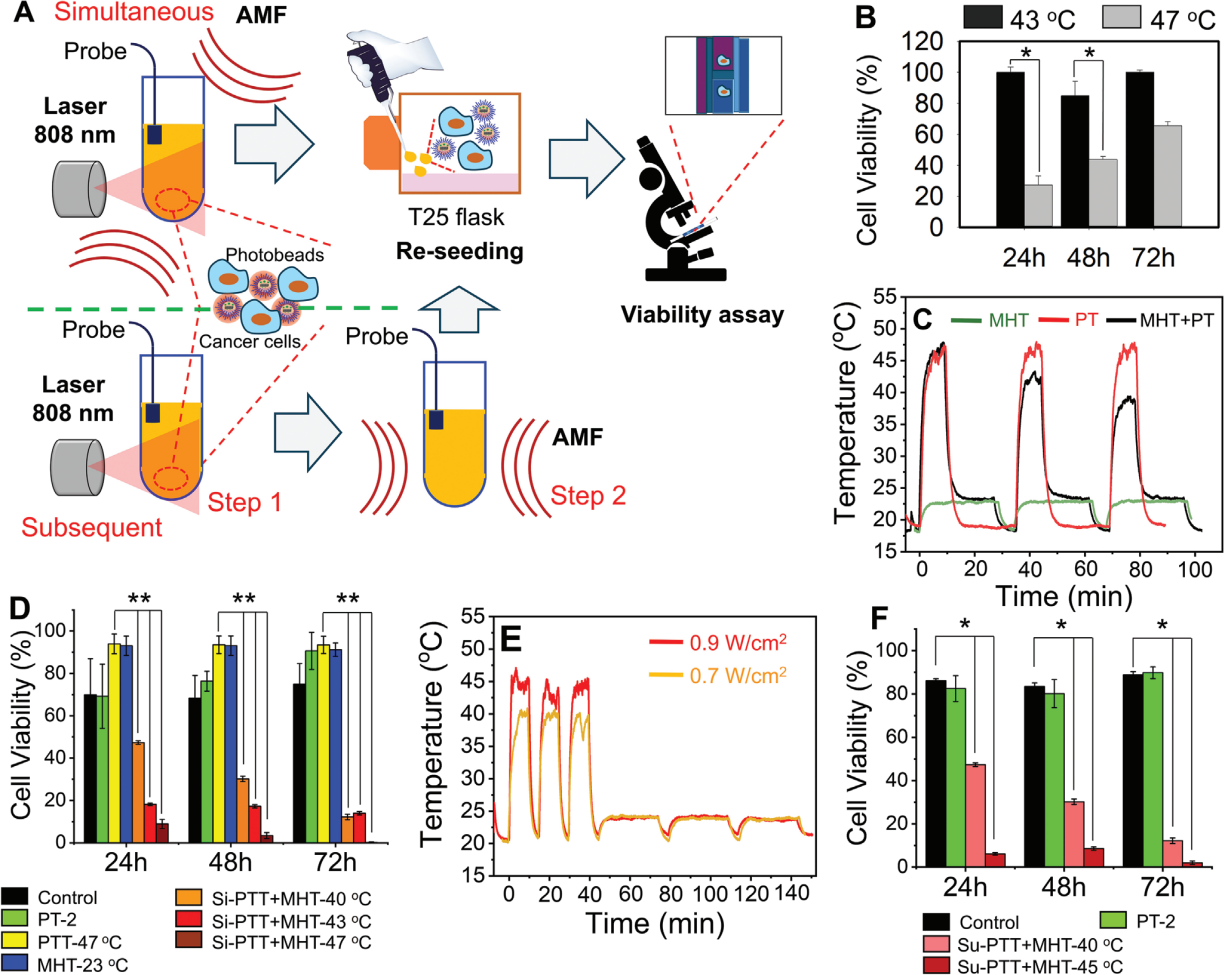
Photobeads as dual MHT and PTT agents. A) Scheme of in vitro efficacy experiment using PT‐2 photobeads to treat cancer cells by combining MHT and PTT following either simultaneous (Si) or subsequent (Su) treatment. B) Cell viability experiment in which U87 cells were exposed to thermal bath set at 43 and 47 °C for a similar time exposure of dual PTT+MHT experiments. Even at the highest temperature of 47 °C, the cells recovered in viability after 72 h, indicating the heat‐resistant characteristic of U87 cells. Values are presented as means with error bars indicating the standard deviation (SD) for n = 3 independent experiments. Statistical analysis was performed using one‐way ANOVA with a Tukey´s post hoc test. *p = 0.015. C) Heating profiles of cell pellets and PT‐2 under PTT only (1.0 W cm^2^, red) or MHT only (16 kA m^−1^ and 282 kHz, orange) or simultaneous exposure to MHT (30 min each cycle at 16 kA m^−1^ and 282 kHz followed by 5 min interval) and PTT (0.9 W cm^2^) (deep red). The maximum temperature of 47 °C was reached in the case of exposure to PTT alone or PTT+MHT. D) Viability results of U87 GBM cells subjected to different conditions: PT‐2 (green bar), PT‐2 + MHT only (blue bar), PT‐2 + PTT only (yellow bar), PT‐2 + dual treatment applied simultaneously (Si‐PTT+MHT). To compare the effect of therapeutic temperature on cell death, the maximum temperature to reach by dual treatment was set to be 40, 43 and 47 °C. In all Si‐PTT+MHT cases, viability less than 15% was recorded after 72 h. Values are presented as means with error bars indicating the standard deviation (SD) for n = 3 independent experiments. Statistical analysis was performed using one‐way ANOVA with a Tukey´s post hoc test. **p = 0.007. E) Heating profiles of the cell pellets treated with PT‐2 and exposed to 0.9 W cm^2^ laser reaching 45 °C (orange curve) or exposed to 0.7 W cm^2^ reaching a maximum temperature of 40 °C (red curve) followed by the MHT application reaching, in this case, a temperature of 23 °C. F) Viability results of U87 GBM cells that are subsequently (one after the other) treated by PTT and MHT (Su‐PTT+MHT) reaching a maximum temperature of 40 or 45 °C (very similar but not exactly the same temperature reached for the Si‐PTT+MHT). Values are presented as mean with error bars indicating the standard deviation (SD) for n = 3 independent experiments. Statistical analysis was performed using one‐way ANOVA with a Tukey´s post hoc test. *p = 0.024.

Provided by the high SAR values under clinical conditions of PT‐2, along with a consistent heating performance in viscous media, and the high photo‐thermal heat profile, to test the effect of dual MHT and PTT hyperthermia, PTT and MHT treatments were applied to tumor cells either in concomitance or one treatment after the other with the PTT applied before the MHT (**Figure** **5**) followed by 2D cell study. To this aim, two millions of tumor cells either the U87 MG or the A431 cultured in flasks, were detached and the cell pellet was suspended in a small volume of PT‐2 solution placed into a glass tube (d = 3 mm). As shown in the scheme of Figure 5A, for the concomitant treatment, the glass tube was exposed simultaneously to 3 cycles of MHT (30 min each with 5 min interval between two consecutive MHT cycles) and 808 nm‐laser exposure (1 W cm^2^) was applied only during the first 10 min of each MHT cycle (to gain more detail on the sample holder for the dual treatment please refers to Figure S5, Supporting Information). The dual treatment was compared to cells exposed only to laser PTT irradiation or MHT under the same conditions applied for the dual treatment. To monitor the damaging effects of MHT, PTT and the dual process, after treatment, the as‐treated cells were re‐cultured in dishes (T25 flasks) and the cell viability was monitored overtime (at 24, 48, and 72 h) by means of trypan blue viability assay.

For U87‐MG cells, given their known heat‐resistant,^[^ ^11^, ^21^ ^]^ and to further verify the heat‐resistant feature of U87 cancer cells to the temperature values selected by us for the therapeutic experiments, we first treated the cell pellets with a classical water bath set at 43 and 47 °C for 3 cycles of 10 and 5 min of interval as done during our dual hyperthermia treatment. Indeed, the experiment at 43 °C did not result in any mortality after 72 h while the cells exposed at 47 °C induced only 35% of cell mortality after the same duration (Figure 5B).

Therefore, in the next step, we first aimed at reaching during dual hyperthermia a therapeutic temperature of 47 °C. Here, we found that using a rather mild laser power of 0.9 W cm^2^ and AMF conditions (respecting biological limit of 16 kA m^−1^ and 282 kHz), the temperature of photobeads solution at a relative low dose (3.6 mgFe mL^−1^) could be raised from 19 to 47 °C within the first 3 min of simultaneous exposure to PTT and MHT and this temperature remained stable at 47 °C during the last 7 min of laser irradiation (Figure 5C). Instead, after switching off the laser while maintaining only the AMF, a sudden drop of temperature of ≈23 °C was recorded, which then remained stable during the last 20 min of MHT treatment. When repeating the second and third cycles of concomitant MHT and laser irradiation, the maximal temperature reached was slightly lower (42 °C and 38 °C for second and third cycle, respectively), while the temperature reached during MHT after switching off the laser irradiation, remained unchanged at all the cycle, indicating that the heating performance of PT‐2 in MHT are not affected by the concomitant application of PTT at least for the first three cycles of MHT while, on the contrary, the lower temperature reached at each of the cycle during dual MHT and PTT, may be related to a partial degradation of the dye.

Interestingly, cells treated under these dual conditions showed a viability of ≈9.5% after 24 h and a complete mortality after 72 h (Figure 5D, deep red bar). For the sample treated only with PTT laser (laser power of 1.0 W cm^2^) when reaching the same therapeutic temperature of 47 °C (see Figure 5C, red curve for the heat profile), surprisingly, the PTT treatment alone did not significantly induce cell death as a viability higher than 90% was recorded at the different incubation times (Figure 5D, yellow bar). The same observation was made when MHT (16 kA m^−1^ and 282 kHz) was used alone although in this case the rise in temperature was of only 5 °C (see the heat profile in Figure 5C, orange curve) and it has mostly no effect on the cell viabilities (>90%) as shown in Figure 5D, blue bar. These data indicate that the temperature values reached is not the only factor involved in the mechanism of cell death and a synergistic effect in this case of combined MHT and PTT is needed to treat U87 cells. To note that for the untreated cells (control) and the cells incubated with PT‐2 but not exposed to any treatment, the viability did not go lower than 70% at 24 h and on the same samples the cell viabilities were higher than 80% a 72 h after treatment, indicating a full cell recovery. It is worth noting that the acute toxicity recorded for the untreated control cells resulted from the experimental conditions: indeed, the control cells were left in a pellet form at room temperature for the same duration of the hyperthermia treatment, and this has caused a transient stress, which upon re‐plating the cells into monolayers, was then recovered.

Then, we investigated whether a lower therapeutic temperature reached during dual MHT and PTT would result in a comparable toxicity effect. Reducing the laser power to 0.8 W cm^2^ while keeping the same MHT conditions enabled us to reach a therapeutic temperature of 43 °C (see heat profile in Figure S10, Supporting Information). Interestingly, this condition in the case of dual treatment resulted in less than 15% of cell viability at 72 h (Figure 5D, red bar). Strikingly, even a temperature as low as 40 °C (0.6 W cm^2^ of laser conditions and the same MHT conditions with the heat profile shown in Figure S11, Supporting Information) induced 50% cell death at 24 h and 15% of cancer cells viability at 72 h (Figure 5D, orange bar).

We also wondered about the effect of PTT and MHT when applied one after the other rather than in concomitant. To test this modality of treatment, the cell pellet with PT‐2 was first exposed to laser reaching 45 °C (0.9 W cm^2^ for 10 min) and subsequently to 3 cycles of MHT (same condition as described above) during which a temperature raise of 4 °C (from 20 to 24 °C) was recorded during each MHT cycle (Figure 5E). Also in this case, the primary PTT treatment did not hamper the MHT performance of photobeads as they always reached the 4 °C increase at each cycle. Significantly, with the dual treatment applied one after the other, a nearly complete cell mortality (viability of ≈5%) was achieved after 72 h of incubation (Figure 5F). The efficacy of this treatment was also studied at a lower therapeutic temperature. Indeed, by setting the laser at 0.7 W cm^2^ the temperature reached during PTT was 40 °C followed by MHT at same field conditions and at a temperature of 24 °C. This treatment condition also resulted in a cell viability of 45% after 24 h and 10% at 72 h of incubation, respectively, being comparable to the case when combining MHT and PTT simultaneously. These data suggest that the subsequent treatment, similar to the simultaneous dual PTT and MHT treatment, does not compromise the therapeutic efficacy, but we would chose the application of one treatment after the other, because it simplifies the instrumentation and reduce the complexity of the experiment.

We further extended this combined MHT and PTT with PT‐2 to another type of cancer cells, namely epidermoid carcinoma A431 to verify the results on another tumor cell type. Here, the subsequent combined treatment was set at a therapeutic temperature of 40 °C being the A431 cells more sensitive to temperature than U87 cells (see Figure S11, Supporting Information for the heat profile). Also for the A431 cells, only the dual subsequent PTT and MHT treatment enabled us to achieve a significant mortality (viability less than 20% after 72 h) while the standalone MHT or PTT resulted in a marginal mortality (viability ≈80%) over the same time window although the therapeutic temperature reached was of only 40 °C(Figure S12, Supporting Information).

Overall, these observations further validate the efficacy of combined MHT and PTT using our photobeads and suggest how toxic effects derived only by the combination of the treatments and not by the absolute therapeutic temperature reached during the treatment.

### 2.6 Elucidating Possible Mechanisms of Cell Death

**Figure 6:**
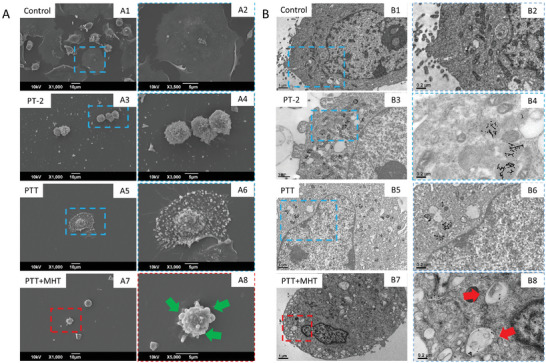
Comparative SEM and TEM morphological characterizations of cells undergoing different treatment conditions at low A1, A3. A5 and A7 and B1, B3, B5 and B7) and high A2, A4, A6 and A8 and B2, B4, B6 and B8) magnification. A1‐2) SEM images of control untreated U87 cells. Dashed blue box A1) is indicating a detail of a cell at higher magnification A2); A3‐A4) SEM images of U87 cells treated with PT‐2 (NPs) materials; A5‐A6) SEM images of cells undergoing only PTT. Dashed blue box A5) indicates a detail of a cell at higher magnification (A6); (A7‐A8) SEM images of cells undergoing PTT and MHT one after the other. Dashed red box A7) is indicating a detail of a cell at higher magnification A8). The round shape cells as well as the round bumps visible at high magnification (green arrows) on the cell membrane indicate the ongoing process of apoptosis cell death. B1‐B2) TEM images of control untreated U87 cells, B3‐B4) TEM images of U87 cells treated with PT‐2 (NPs) materials; (B5‐B6) TEM images of cells undergoing only PTT (B7‐B8) TEM images of cells undergoing PTT and MHT one after the other. In this experiment, some U87 cell pellets were exposed to PT‐2 ([Fe] = 3.6 g L^−1^) and treated with only PTT (laser 808 nm, 1.0 W cm^−2^, reaching 45 °C) (dashed blue boxes are indicating cell details at higher magnification), or a subsequent PTT and MHT treatment (PTT under an 808nm‐laser at 1. 0 W cm^−2^, reaching 45 °C and MHT at 16 kA m^−1^, 282 kHz, reaching a temperature of 25 °C) (dashed red boxes are indicating a detail of a cell at higher magnification and red arrows are highlighting the presence of endocyted photobeads into cells). SEM images were taken at 1000X magnification for low magnification images and 3000X magnification for high magnification images. TEM pictures were taken at10 000X magnification for low magnification images and 25 000X magnification for high magnification pictures.

To investigate the possible mechanism that accounts for such high cell mortalities in the dual treatment, we performed cellular morphological study by Scanning Electron Microscopy (SEM) and Transmission electron microscopy (TEM) of cells treated in different conditions (**Figure** **6**A and B respectively). We compared the cells morphology when exposing U87 cells to the subsequent PTT+MHT dual treatment reaching 45 °C as therapeutic temperature, or to PTT alone, at the same therapeutic temperature of 45 °C: These data were also compared to controls cells that were not exposed to any materials or to U87 cells that were treated with PT‐2 photobeads. Cells treated with dual MHT and PTT exhibited clear signs of suffering as they are quite roundish and presented a high number of vesicles, bubbles and protrusions compatible with apoptotic bodies and which are clearly missing in the control untreated cell samples in which cells are well spread and adhering at the substrate (Figure 6 panels A1‐A2). Some cell damage signs were observed also after treatment with PTT when reaching the same 45 °C temperature (Figure 6A5‐A6). However, in this case, although many bubble/like bodies were present on the cells indicating some signs of cell sufferance, cells were not as roundish as in the case of dual therapy, while cells appear still adhering at the substrate and having filipodia of adhesion to the substrate, a distinct sign not observed on the combined treatment of PTT+MHT. Cells treated with PT‐2 materials only (Figure 6A3‐A4), exhibit a rounded morphology with no evidence of vesicles, bubbles and protrusions compatible with apoptotic bodies as observed in PTT+MHT experimental condition. On PT‐2, few signs of suffering are observed (probably due to slightly toxic effect of nanoparticles and hypoxic conditions through the treatment). However, on higher magnification (Figure 6 A4) the extension of filopodia which enable the cells to adhere to cell substrate, represents also a sign of cell spreading and migration. In the dual therapy, after PTT although the MHT does not affect the temperature reached it may provoke other local toxic effects having a more local nature.

Indeed, it may also occur that cell damage signs are also present intracellularly. For that reason, TEM analysis was performed on the same cell samples after exposure to the different treatments (Figure 6B). From the direct comparison of cells exposed to dual treatment and to PTT only at the same therapeutic temperature of 45 °C, again clear signs of cell damage were observed for the dual PTT and MHT treatment with a clear de‐structuring of the cytoskeleton, granular/like structure of the cytoplasm and the presence of numerous apoptotic bodies, the empty vesicles close to the cells membrane (Figure 6 B7 and B8). The intracellular structure of PTT sample at the same therapeutic temperature of 45 °C (Figure 6 B5 and B6) did not look as damage as the dual treatment, since no granular structure of the nucleolus could be observed in this case. For both dual PTT and MHT and PTT samples the presence of dark contrast nanocubes within endosomal‐like vesicles indicated the uptake of the PT‐2 beads by the cells. These data suggest how the addition of MHT to the PTT changed significantly the local toxicity on cells although MHT alone just reach 25 °C. To further complete the intracellular effect, the TEM of cells treated with photobeads and exposed to only MHT, but which did not receive any PTT treatment were also collected (Figure S13, Supporting Information). These TEM images clearly indicated that the beads alone (Figure 6 B3‐B4) or the MHT alone at 25 °C (Figure S13, Supporting Information) were not toxic to cells as the cell did not change their microstructures and cytoskeleton structure when compared to control cells, although the presence of beads were observed in all these samples in endosomal‐like vesicles. PT‐2 materials at 24 h are mainly internalized within subcellular vesicular and perinuclear structures which, provided the time of exposure, are compatible with late endosomes or lysosomal vesicles (Figure S12, Supporting Information).

Next, to deeply investigate the local toxic effects on cells, we were wondering if internalization of our nanobeads under the exposure to dual PTT and MHT treatment could induce toxicity effects related to permeabilization of lysosomes membranes. The endosome permeabilization effect has been documented in some hyperthermia treatment, mostly due the permeabilization of lysosomal membrane and consequent release of lysosomal proteases in the cytosol, causing digestion of vital proteins and the activation of hydrolases including caspases.^[^ ^7^, ^66^, ^67^, ^68^ ^]^ The combination of permeabilized lysosomes, together with increased temperature in the combined treatment (PTT+MHT), can cause the disorganization of the cytoskeleton observed only for dual therapy (in Figure 6B) in comparison with the rest of applied treatments in which said de‐structuring of the cytoskeleton was not observed.

**Figure 7:**
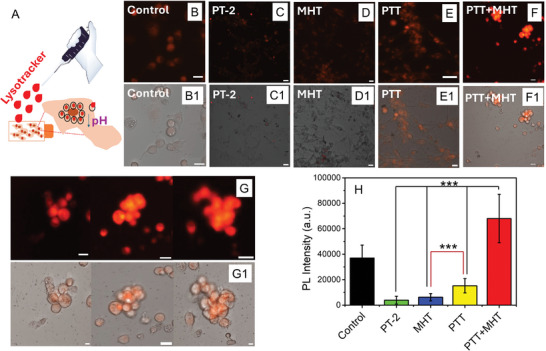
Investigating lysosomes permeability via lysotracker assays. A) Upon exposure to different treatments cells were incubated with lysotracker, a dye that turns to be more fluorescent in response to acidic lysosomal pH. Confocal images B–G) and confocal and bright field merged channels B1–G1) of cells differently treated: control cells B/B1), cells treated with PT‐2 photobeads only C/C1), cells treated with PT‐2 photobeads and undergoing MHT D/D1), cells treated with PT‐2 photobeads and undergoing PTT E/E1) and cells treated with PT‐2 photobeads and undergoing dual PTT+MHT one after the other F/F1 and G/G1), respectively. The cells group undergoing dual PTT+MHT has a bright fluorescent signal which appears spread all over the cell body as a clear indication of the lysosomal disfunctions F/F1 and G/G1). From B/B1 to F/F1 magnification of 10X. Panels G/G1 are a detailed magnification (20X) of treated cells with the combined therapy PTT+MHT. Scale bar: Control B/B1 20 µm; NPs C/C1: 50 µm; MHT D/D1: 20 µm; PTT E/E1: 20 µm; PTT+MHT F/F1: 20 µm. H) Quantitative PL intensity signal analysis of different cell groups after staining with lysotracker. Images for the PL analysis quantification for each of the groups (Control, PT‐2, MHT, PTT, PTT+MHT) were taken under the same confocal experimental conditions (20X magnification and under the same confocal setting parameters). PL intensity represents the mean value of three independent experiments with error bars indicating the standard deviation (SD). Statistical analysis was performed using one‐way ANOVA with a Dunn´s post hoc test. ***p = <0001. Black asterisks indicate statistical differences found in PTT+MHT versus PTTs experimental conditions. Red line shows statistical differences between PTT versus MHT experimental conditions.

For the lysosomal permeabilization test, U87 cells, after exposure to PT‐2 only or with exposure to the different hyperthermia treatments, were kept in incubation for 24 h, prior to being exposed to a pH‐sensitive fluorophore which, becomes more fluorescent in acidic media (lysotracker), and lately subjected to confocal imaging analysis. Remarkably, in comparison to all the other groups (**Figure** **7**, panel B, C, D, E, and F) only cells undergoing the combined MHT and PTT featured a much higher red fluorescent signal which was spread all over the cell cytoplasm rather than appearing more punctiform and confined within the lysosomes compartment as it should appear in physiological cells. This confirms the permeabilization of lysosomal membranes with subsequent leakage of lysosomal hydrolases into the cytosol only for the dual PTT and MHT sample (Figure 7F/G and F1/G1). The spreading of lysotracker signal on the combined treatment sample, as observed in Figure 7G and G1, fully agrees with lysosome membrane disruption, as also reported by other research groups.^[^ ^69^ ^]^

A quantitative count of fluorescent intensity signal normalized to each cell by ImageJ analysis revealed how cells treated with combined dual PTT and MHT treatment have the highest red PL intensity with PL intensity values being almost one order magnitude higher than those of the other groups (Figure 7H). For each group at least 500 cells were considered. Lysotracker staining experiments, also performed on epidermoid carcinoma cells (A431) also revealed a similar trend with the highest fluorescent signal again recorded in case of dual treatment in comparison to other groups (the single therapy groups, Figures S14 and S15, Supporting Information). Taking these results together, we suggest that the combined PTT and MHT using photobeads NPs might induce the cell death by means of lysosomal permeabilization and subsequent apoptosis induction. Previous reports have already connected lysosomal proteases released from the lysosomes into the cytosol as a factor to induce an apoptotic cascade mediated by mitochondria.^[^ ^69^ ^]^ Also, it has been reported that massive lysosomal rupture induces the release of the entire contents of the lysosome, triggering a cascade of hydrolysis of the cytoplasmic content.^[^ ^70^ ^]^ At the same time, the presence of many apoptotic bodies in TEM images taken from cells treated with PTT+MHT (Figure 6) suggests the possibility of cell death by apoptosis induced and here mediated by lysosomal membrane permeabilization.

Particularly remarkable is that this effect was more pronounced on the dual treatment not because of the pure temperature reached during the treatment but because of local hot spot effects or other local effects which were occurring at the nanobeads overcoming the heat resistance of these cells and inducing concomitant micro toxic cell damage effects.

## 3 Conclusions

We here report the development of novel multifunctional magnetic photobeads for cancer treatment by means of combined of photothermia (PTT therapy) and magneto‐thermia (MHT). An amphiphilic diblock copolymer made of hydrophilic PEG and hydrophobic poly(methacrylamides) having diol and benzyl side groups was synthesized by a scalable three‐step synthetic procedure based on the photo‐atom transfer radical polymerization of an activated ester methacrylate, its aminolysis reaction with furfuryl amine and the subsequent furfuryl‐maleimide click chemistry to introduce the benzyl side groups. The developed polymer was used to co‐encapsulate magnetic nanoparticles, as superior heat mediators for MHT, and IR780 dye, as efficient photothermal and NIR‐imaging agent. By means of a simple and scalable self‐assembly protocol, the optimal conditions to maximize encapsulation of IR780 dye (72 µg mgFe^−1^) and iron oxide nanocubes into well‐defined magnetic photobeads in which chains of IONCs are formed during encapsulation and a considerably high amount of IR780 were well‐encapsulated in compact polymer beads. Interestingly, the photobeads feature high MHT heat performances measured in terms of SAR values, being comparable to the ones of PEGylated IONCs having a similar edge size, under MHT field conditions of clinical use. Remarkably, even if the nanocubes were encapsulated in the polymer beads, the heating capacity of photobeads remains unchanged even in a high viscous media, highlighting the potential of photobeads usage as heat mediators in tumor and intracellular microenvironment, as here shown. On temperature resistant cell populations such as Glioblastoma U87 cell line, exposing the cells to these beads and to dual PTT and MHT, either simultaneously or sequentially, resulted in cytotoxic effects that were not observed with single MHT or PTT treatments. To note, the dual toxic effects were studied at different therapeutic temperatures of 47, 45 and 40 °C and the viability cell data obtained confirmed the remarkable difference of the dual treatment versus single treatment even if, the PTT alone was reaching the same therapeutic temperature of respectively 47, 45 and 40 °C.

By SEM, TEM and confocal analysis, we found that only on the combined PTT and MHT (and not in case of only PTT at the same therapeutic temperature or when applying only MHT) resulted in an unconventional mechanism of cell death most likely based not on the pure thermal damage effect but on microstructural cell damage. Indeed, on cells treated with PTT+MHT the structural morphology of apoptotic round shaped cells, the presence of many apoptotic bodies on each cell visible in SEM images (Figure 6, green arrows) and TEM images suggest the induction of cell death by apoptosis induced by lysosomal disfunction. On the other hand, apoptosis has been connected to lysosomal membrane permeabilization as previously reported.^[^ ^69^, ^70^ ^]^

This, in turn, led to the possibility to eradicate different types of cancer cells including temperature‐resistant cells even by a short duration exposure to a relative mild temperature as low as 40 °C. Our magnetic photobead‐based approach by integrating PTT and MHT represents a step ahead for dual cancer therapy thanks to the peculiar synergic effects we observed at mild temperature (41–43 °C) and only when applying PTT and MHT together. Previous studies have explored magnetic based platforms for magnetically guided phototherapy or have used bare lipidic nanostructures for the delivery of only IR‐780,^[^ ^71^ ^]^ but the mechanism of action of our dual of PTT and MHT based on magnetic nanoparticles and IR‐780 dye of our photobeads is distinct and not previously reported in the literature.^[^ ^72^ ^]^ It is worth mentioning that while magnetic hyperthermia can be applied remotely, PTT requires the NIR‐laser which poses some tissue penetration concerns and can be applied only to superficial tumours. However, new technological tool like tapered fibres or waveguide, so far used to deliver the light for optogenetic experiments in brain of mouse, may be soon adopted, as tools to deliver the laser light in deep tumour and activate the photo‐responsive nanoparticles thus making feasible this type of dual PTT and MHT therapies.^[^ ^73^, ^74^, ^75^ ^]^ Due to the great potential of this novel nanomaterial, soon, we will aim at investigating more in deep, the in vitro action of these photobeads, analyzing lysosome membrane disruption mechanisms by more specific assay, even considering potential DNA damage, cell cycle arrest, more specific and other cellular stress responses Furthermore, we will validate in a preclinical animal model their efficacy on a xenograft tumor model.

## Conflict of Interest

The authors declare no conflict of interest.

## Author Contributions

B.T.M., T.F. and T.P. conceived the idea and designed the experiments. H.G.R prepared the IONCs. B.T.M, J.S.C and L.G synthesized the polymers and performed the self‐assembly experiment to obtain photobeads. B.T.M. and J.S.C. performed the characterizations of obtained materials. S.F. helped with measuring the heating profile of photobeads under combined PTT+MHT condition. T.F.C, G.N. carried out the cell viability study. T.F.C performed the lysosomal permeabilization test, confocal analysis and the quantitative analysis of fluorescent signal. T.F.C prepared the samples for microscope study and D.D. carried out the microscope study both (SEM and TEM imaging). B.T.M., T.F.C, J.S.C., G.N. and T.P. were involved in data analysis. B.T.M. and T.P. drafted the paper. All authors read and corrected the paper.

## Supporting information



Supporting Information

## Data Availability

The data that support the findings of this study are available from the corresponding author upon reasonable request.
