# Supplementary material for: Nanoplatforms for Magnetic‐Photo‐Heating of Thermo‐Resistant Tumor Cells: Singular Synergic Therapeutic Effects at Mild Temperature
Source: Small. 2024 Oct 28;20(51):2310522. doi: 10.1002/smll.202310522 (PMC11657026; doi:10.1002/smll.202310522)
Supplement: Supplementary file 1 — Supporting Information [file SMLL-20-2310522-s001.docx]

**Nanoplatforms for Magnetic-Photo-heating of Thermo-Resistant Tumour Cells: Singular Synergic Therapeutic Effects at Mild Temperature**

Binh T. Mai*^¥1^, Tamara Fernandez-Cabada ^#1^, John S. Conteh^1^, Giulia E.P. Nucci^1^, Sergio Fiorito,^1^ Helena Gavilán^&1^, Doriana Debellis^1^, Lorenci Gjurgjaj^1,2^ and Teresa Pellegrino*^1^

1. Italian Institute of Technology, via Morego 30, 16163, Genoa, Italy
2. The Open University Affiliated Research Center, Italian Institute of Technology, via Morego 30, 16163, Genoa, Italy

*Corresponding authors: [binh.mai@universityofgalway.ie](mailto:binh.mai@universityofgalway.ie) [Teresa.Pellegrino@iit.it](mailto:Teresa.Pellegrino@iit.it)

**Experimental section**

**Materials for the synthesis**

IR-780 Iodide (Merck, ≥ 95%), Triethylamine (TEA, Merck, 99%), (±)-3-Amino-1,2-propanediol (APD, Merck, 97%), Furfuryl Amine (FA, Merck, ≥ 99%) and Benzyl Maleimide (BM, Merck, 99%) were purchased and used as received. All the chemicals were stored at ambient condition. N-succinimidyl methacrylate (NSMA) was synthesized by the esterification between N-hydroxy succinimide (NHS) and methacryloyl chloride as described elsewhere in the literature.^1^ Polyethylene glycol functionalized with ATRP initiator (PEG-Br) and 2-bromo-N-(3,4-dihydroxyphenethyl)-2-methylpropanamide (DOPA-BAmBr) were synthesized following the procedure in our previously reported studies.^1, 2^ All the solvents were purchased from commercial sources with the highest available purity, and they were used as received. Iron oxide nanocubes IONCs (edge size 14 nm) was synthesized *via* the solvothermal technique as reported in our previous study.^3^

**Materials for the Cell culture**

A431 epidermoid carcinoma cells (ATCC CRL1555) was purchased by ATCC Company. U87-MG cell line (ATCC® HTB-14™) was kindly provided by Dr. Emilio Ciusani from Carlo Besta Neurological Institute from Milano (Italy). The cells were cultured in Dulbecco’s Modified Eagle’s Medium (DMEM, high glucose) (D5671, Sigma Aldrich), supplemented with 10% fetal bovine serum (heat inactivated FBS), 2% penicillin−streptamycin (10000 U/mL), and 1% L-glutamine (200 mM) at 37 °C, 5% CO2, and 95% relative humidity on T75 flasks (Corning®). All of the cell culture reagents were purchased from Gibco.

**General Characterization**

^1^H NMR spectra were recorded using a Bruker 400 MHz BBI spectrometer. Deuterated DMSO was used as a solvent at 25°C for all the measurements. Size Exclusion Chromatography (SEC) was performed to investigate the molar masses of polymers. SEC measurements were carried out on an Agilent 1260 Infinity quaternary LC system consisting of an Agilent 1260 Infinity quaternary pump (G1311B), autosampler (G1329B), two PLGel 5μm MIXED-C columns (kept at 25 °C) and a refractive index detector (G1362A). DMF containing LiBr (10 mM) was used as an eluent at a flow rate of 1 mL/min. The molar masses were determined using Agilent narrow molecular mass distribution polystyrene standards in DMF (LiBr 10 mM). The particle sizes were characterized by dynamic light scattering (DLS) using a Malvern Instruments Zetasizernano series instrument. Samples were diluted to reach a solution having [Fe] of 0.1 mgFe/mL. An equilibration time of 1 min was allowed prior to each reading, and at least three replicate measurements were made for each sample.

Transmission Electron Microscopy (TEM) images were obtained using a JEOL JEM 1011 electron microscope, equipped with a W thermionic electron source and a 11Mp Orius CCD Camera (Gatan company, USA), with an acceleration voltage of 100 kV. The samples were prepared by placing a drop of the sample onto a carbon coated copper grid, which was then left to dry before being subjeted to the microscope.

Elemental analysis was carried out via Inductively Coupled Plasma (ICP) Atomic Emission Spectroscopy on a ThermoFisher CAP 6000 series. The samples were prepared by digesting 10 μL of the sample in 1.0 mL of aqua regia overnight, followed by dilution with Milli-Q water to 10 mL. This solution was filtered through an PTFE membrane (0.45 μm) before the analysis.

Visible-Near InfraRed (NIR) absorption spectra were recorded on a Varian CARY-Eclipse 50 Scan UV–visible-NIR spectrophotometer. The Visibile-NIR absorbance measurements were carried out in the wavelength range of 500-900 nm. PhotoLuminescent (PL) spectra were measured using a Cary500 eclipse spectrometer, and the excitation wavelength was 750 nm. For all measurement, samples were diluted to reach a solution having [Fe] of 0.1 mgFe/mL. The total volume of measured samples was 3.0 mL.

**SAR measurement**

The specific adsorption rate (SAR) values of samples were measured in water and in glycerol (15%, 36%, 60%, 81%) using a commercially available DM 100 Series Nanoscale Biomagnetics Corp.) setup. A 150 μL of each photobeads sample ([Fe] ≈ 2.5 ~ 4.0 g/L) was introduced to the instrument holder and exposed to alternating magnetic fields at different frequencies and magnetic field intensity. The exposure time was approximately 60 s, and all measurements were repeated four times. The SAR values of all the samples were calculated following the formulation below:

SAR (W/g) = $\frac{C}{m}\times\frac{dT}{dt}$

in which C is the specific heat capacity of dispersed media and m is the concentration of Fe (g.L^-1^) in solution. The measurements were carried out in close-to-adiabatic conditions, thus the slope of the curve dT/dt was determined considering only the first few seconds. For the measurement in viscous media, the specific heat capacity of glycerol solution at different concentrations of glycerol was used as reported in our previous study.^4^

**MHT coupled with Photothermal treatment measurement**

50 μL of photobeads (PT-2, [Fe] = 2.5 g.L^-1^) was loaded in a capillary tube having a diameter of 3 mm and length of 25 mm. The tubes was then placed at the center of the coil of magnetic hyperthermia device using a home-made holder. The temperature probe (optical fiber) was then inserted and adjusted to touch the upper part of solution in order to avoid its direct exposure to laser which will create the interference of temperature readout. Afterwards, the tube was exposed to three cycles of laser irradiation (7 minutes each, wavelength of excitation ( λ ext) 808 nm, 4.67 W/cm^2^) along with 5 minutes of interval. After three laser treatment cycles, the sample was simultaneously exposed to the same laser irradiation and an AMF (24 kA/m and 120 kHz) for another 7 min. For this experiment, the MHT was performed on D5 Series Nanoscale Biomagnetics Corporation. The open coil of this device enables us to apply laser and AMF simultaneously or subsequently as shown in Figure S4.

**Confocal image**

For PT-2 nanomaterial intracellular localization, U87-MG cells 3D model were cultured in suspension, on CorningT25 ultralow attachment flasks (Sigma Aldrich) and treated with 3.18 gFe/L of PT2 magnetic-Photobeads for 24 and 48h. Photobeads were dispersed in DMEM/F12 medium supplemented with 100 μg/mL penicillin/streptomycin, 1% B-27 supplement (Gibco, Thermo Fisher Scientific, Waltham, MA USA), 10 ng/mL basic FGF and 20 ng/mL EGF (Peprotech EC, London, UK). After the due incubation time, the cells were washed three times with PBS 1X pH 7.4 and fixed with 4% paraformaldehyde. The plate was viewed under a confocal microscope (A1+ confocal microscope system, Nikon) at an excitation/emission wavelength of 745± 35 nm for IR780 dye.

***In vitro* anticancer efficacy by combined MHT and PTT**

For the MHT and PTT experiment and cell viability study, 2*10^6^ cells were incubated with 60 ul of 3.18 g/L of PT2 Photobeads dissolved in DMEM, high glucose, phenol red free, supplemented with 10% fetal bovine serum (heat inactivated FBS), 2% penicillin−streptomycin (10000 U/mL), and 1% L-glutamine (200 mM) and were placed inside a capillary tube with a diameter of 3 mm and length of 25 mm. The tube was placed inside a sample holder in the middle of the coil of magnetic hyperthermia device as shown in Figure S3. PTT (808 nm laser at 0.6 – 1.1 W.cm^-2^ for 10 min; 3 cycles of 10 min) and/or MHT (282 kHz and 16 kA/m 3 cycles 30 min/cycle) were applied while the temperature increase was monitored by the temperature probe (optical fiber) inserted inside the capillary tube. At the end of the treatment, the cells were recovered by centrifugation and diluted in complete DMEM were seeded in T25 flask culture at a density of 6 × 10^5^ cells per flask and allowed to adhere overnight.

24, 48 and 72 hours after the cell re-culturing, cell viability was studied by Trypan Blue assay. For the test, cells were diluted in PBS and in trypan blue solution (0.4%) (1:1 volume) and placed on hemo-cytometer for viability count under the microscope (Fig 4A). Blue cells correspond to dead cells meanwhile colourless cells (brightness) correspond to alive cells. The experiment was done in triplicate (n = 3) for each of the group analyzed, and the mean value was plotted with an error bar to represent the standard deviation. Tested experimental groups correspond to: **CTRL**: Control cells (U87-MG or A431 cell line) treated with complete DMEM and placed at room temperature for a duration time of 2h corresponding to the full experiment duration; **PT2**: cells incubated with 3.18 gFe/L of PT2 Photobeads in complete DMEM medium and placed at room temperature for duration of the experiment and then reseeded for the cell viability study; **PT2 NIR**: cells treated with 3.18 gFe/L of PT2 Photobeads in complete DMEM medium and expose to NIR treated prior to re-culturing for viability assay; **PT2 MHT**: cells treated with PT2 photobeads and MHT treatment prior to re-culturing for viability study; **PT2_(NIR+MHT)**: Cells treated with PT2 Photobeads and treated with first NIR and then with MHT treatment).

**SEM imaging**

For Scanning Electron Microscopy (SEM) experiments, the samples were prepared by seeding 5× 10^5^ U87-MG cells previously treated accordingly to the experimental conditions above reported on a cover slip. Upon adhesion (24 hours at 37 °C), the samples were then fixed with 1.5% glutaraldehyde in a 0.1 M sodium cacodylate buffer for 2h at room temperature (pH=7.4). After fixation time, samples were washed three times with 0.1 M sodium sodium cacodylate buffer and post-fixed with an aqueous solution of Osmium Tetroxide (1% volume) for 2 hours. Subsequently, cells were dehydrated in graded series of water-ethanol solution (from 30%V to 100% volume 10 minutes each), followed by an intermediate step of 1:1 ethanol:hexamethyldisilazane (HMDS, Sigma-Aldrich) and as the last step a 100% volume HMDS with final drying step overnight in air. The samples were then fixed on an aluminium stub and coated with 10 nm of gold. SEM analysis were performed using a JEOL 6490 LA scanning electron microscope operated at 10 KeV of accelerating voltage.

**TEM imaging**

After the treatment, cells were fixed and analysed for transmission electron microscopy (TEM). 1*10^6^ cells were incubated in complete DMEM medium supplemented with glutaraldehyde (2% in volume), for 45 min at room temperature. Cells were then centrifuged at 14,000 rpm for 10 min. The obtained pellet was fixed in a 1.5% in volume glutaraldehyde solution in sodium cocodylate buffer (0.1 M, pH 7.4) for 1 h at room temperature. Afterwards, cells were centrifuged at 14,000 rpm for 10 min and washed in the same buffer. The samples were post-fixed in osmium tetroxide in MQ water (1% in volume ) for 2 h and stained overnight at 4 °C in an aqueous uranyl acetate solution (1% in volume). After several washes in MQ water, the samples were dehydrated in a graded ethanol series and embedded in SPURR resin. Sections of about 70 nm were cut with a diamond knife on a Leica EM UC6 ultramicrotome and images were collected with a JEOL JEM 1011 electron microscope operated at 100 KeV equipped with a Gatan Orius SC1000 series CCD camera.

**Lysosome studies**

24 h after cell treatments, at least 5*10^5^ U87-MG or A431 cells per each tested experimental condition, were stained with a solution of Lysotracker probe (LysoTracker™ Red DND-99, Thermo Fisher Scientific). The probe was diluted in phenol free cell culture medium (2 uM) and incubated at 37°C for 45 minutes. After the incubation time, cells were washed three times with PBS and visualized by confocal microscope at an excitation/emission wavelength of 577/590 nm for Lysotracker red. For quantitative analysis of fluorescence of the confocal images, the threshold of the images was set to HV=88, offset=1, power laser =6.7, At least 300 cells were counted per each of experiment (n=3 independent experiments). Image analysis was performed using Image J software. The area of the cell was selected with the “create selection” tool function and the fluorescence intensity of the selected area was measured, subtracting the background of the image for the correct quantification of fluorescence. Values are represented as mean ± SD.

**Synthesis of reactive PEG-*b*-PNSMA by Photo-ATRP**

The synthesis of poly(ethylene glycol-*block*-N-succunimidyl methacrylate) was carried out by taking advantage of the Photo-ATRP process using ATRP intiator-functionalized PEG (PEG-Br) as macro-initiator. Briefly, 1.0 g of PEG-Br (M_w_ 2000 g.mol^-1^, 0.5 mmol) was weighed in a 40 mL open-capped glass vial, and 4 mL of DMF was added to dissolve the polymer. After that, NSMA (2.75 g, 15 mmol) was added, and the resulting mixture was stirred for a further 15 min. Subsequently, PMDETA (13.6 µL) were added to the solution. The vial was sealed and purged with a nitrogen flow for 10 min. Meanwhile, a stock solution of CuBr_2_ (0.02 mM) in DMF was prepared and purged with a nitrogen flow for 10 min. After, 1.0 mL of this solution was injected into the polymerization vessel containing the monomers, ligand and initiator. The resulting solution was stirred for a further 5 min under nitrogen flow. Finally, the vial was placed under a UV source and kept in the cold room to initiate the polymerization. A nail gel curing lamp (λ_max_ ~ 360 nm) equipped with four 9W bulbs was used as a UV source. After 18 h of irradiation, the viscosity of polymerization solution dramatically increased. This viscous solution was precipitated with an excess amount of acetone (10 folds volume). The precipitate of polymer was collected by means of centrifugation, then it was re-dissolved in DMF. Acetone was added again to precipitate the polymers. This dissolution-precipitation step was repeated twice to remove the unreacted monomers and CuBr_2_ catalyst. The final precipitate was further washed with diethyl ether prior to being dried in a vacuum oven set at 40 ^o^C for 24 h to obtain 2.8 g polymer. The obtained PEG-*b*-PNSMA in form of a glassy solid was characterized by means of ^1^H NMR and SEC.

**Synthesis of PEG-*b*-P(PDMAm-*co*-FMAm) by aminolysis reaction of PEG-*b*-PNSMA**

PEG-*b*-PNSMA (1.0 g), containing 5.35 mmol of NSMA, was dissolved in DMSO (10 mL) for overnight in a 40 mL glass vial. This solution was vigorously agitated by means of magnetic stirring, followed by the addition of 2-aminopropandiol (196 mg, 2.14 mmol) and furfurylamine (760 μL, 8.56 mmol). Successively, TEA (75 μL) was added, and the resulting solution was left stirred for 18 h. After the reaction is done, the polymer solution was diluted with an equal volume of THF (10 mL) and then precipitated in an excess amount (10 folds volume) of diethyl ether. After that, the obtained polymer was dissolved in DMF and then the precipitation in diethyl ether was performed. The polymer was collected by means of centrifugation and the obtained polymer pellet was dried overnight in a vacuum oven set at 40 ^o^C to yield 0.8 g of the final polymer product, PEG-*b*-P(PDMAm-*co*-FMAm), which was subsequently characterized by ^1^H NMR and SEC.

**Synthesis of PEG-*b*-P(DiolMAm-*co*-BenzylMAm) by Diels-Alder reaction**

PEG-*b*-P(PDMAm-*co*-FMAm) (0.5 g, 2.07 mmol of FMAm) was dissolved in 5 mL of DMF in a 40 mL glass vial. To this solution, benzyl maleimide (776 mg, 4.14 mmol) was added and the reaction was kept shaking for 72 h at room temperature. Afterwards, 30 mL of cold diethyl ether was added, and a polymer pellet was obtained by the centrifugation, which was then dissolved in 5 mL of DMF. This solution was precipitated again in diethyl ether. After the centrifugation, the supernatant was discarded and the collected pellet was dried in vacuum oven set at 40 ^o^C for overnight to obtain 0.52 g of PEG-*b*-P(DiolMAm-*co*-BenzylMAm). The final polymer product was characterized using ^1^H NMR and SEC.

**Ligand exchange of IONCs with DOPA-BiBAm**

The ligand exchange was carried out following the procedure reported here below. Namely, 23 mg DOPABiBAm was dissolved in 3 mL of CHCl_3_/MeOH mixture (4% MeOH) in a 8 mL glass vial. To this solution, 180 μL solution of IONCs (edge size 14 nm) capped with oleic acid in CHCl_3_ ([Fe] = 5.0 g.L^-1^) was added and the resulting solution was sonicated for 30 sec. The number of DOPA-BAmBr per nm^2^ was set to be 500. TEA (11.5 μL) was successively added to this solution and the obtained solution was sonicated for 30 sec. The vial was then cover with aluminum foil and left in a orbital shaker set at 800 rpm for overnight. The solution after ligand exchange reaction was added with 5 mL of hexane and centrifuged at 1800 rpm for 10 min. The reddish supernatant was discarded and the brownish pellet was redispersed in 1 mL of THF, followed by the addition of 5 mL hexane. After a centrifugation step (1800 rpm, 10 min), the supernatant was discarded and 250 μL of THF was added to resuspend the IONCs. To this solution, 750 μL of DMF was added to obtain a dark and clear solution which was later used in the self-assembly process.

**Preparation of magnetic photobeads by self-assembly process and quantification of the amount loaded of IR780**

To an 8 mL galss vial, 160 μL of IONCs capped with DOPABiBAm in THF/DMF solution (25% THF) ([Fe] = 0.9 g.L^-1^) was added, followed by the addition of 30 μL solution of PEG-*b*-P(PDMAm-*co*-BMAm) in DMF (100 mg.mL^-1^). The resulting solution was shake well and 3 μL of solution of IR780 in DMF (10 mg.mL^-1^) was added. The vial was then fixed into an orbital shaker set at 1100 rpm and MiliQ H_2_O was added using a syringe pump with a rate of 8 mL.min^-1^ during the shaking. Afterwards, the vial was put to a static magnet (0.3 T) to remove the empty polymer beads that do not contain IONCs. The supernatant was discarded when all photobeads had accumulated at the magnet. MiliQ H_2_O was added and the sample was washed two more times using the same magnetic decantation. The condition reported here is for PT-2. To get other photobeads with different amount of IR780 loaded, the volume of IR780 solution added was change accordingly while all other parameters were kept constant.

To quantify the amount of IR780 loaded, 10 μL of photobeads solution ([Fe] = 3.6 g.L^-1^) was diluted with 90 μL of DMF to induce the disintegration of photobeads and a complete release of loaded IR780. IONCs was precipitated out using a high-speed centrifugation (5000 rcf) and the supernatant containing IR780 and polymer was subjected to the optical absorption spectroscopy. Owing to its characteristic absorption peak at 780 nm, the concentration of IR780 in solution can be determined using a calibration curve of IR780 in H_2_O/DMF mixture (10 % H_2_O).


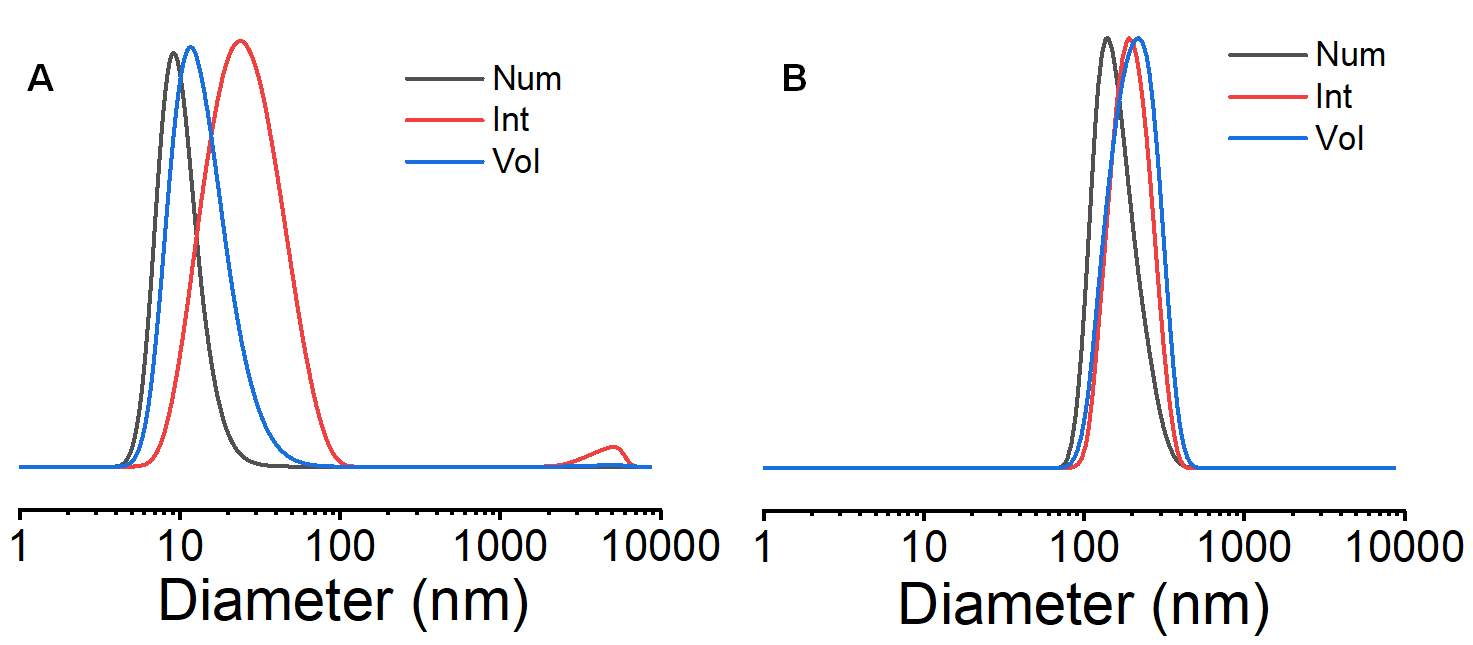


**Figure S1.** DLS traces of fully soluble PEG-*b*-P(PDMAm-*co*-FMAm) (A) and self-assembled nanostructure of PEG-*b*-P(PDMAm-*co*-BMAm) (B) in water. As PEG-*b*-P(PDMAm-*co*-FMAm) is fully water soluble, the polymer is dissolved in water to form single chain (not self-assembled nanoparticles) that have a very small d_H_.

**Table S1.** Hydrodynamic size (d_H_) and polydispersity index (PDI) of photobeads obtained when changing the feeding amount of IR780 solution.

| Sample | Weighted by number (nm) | Weighted by intensity (nm) | Weighted by volume (nm) | PDI |
| --- | --- | --- | --- | --- |
| PT-0 | 90 ± 6 | 140 ± 4 | 126 ± 2 | 0.098 |
| PT-1 | 133 ± 8 | 196 ± 1 | 206 ± 2 | 0.116 |
| PT-2 | 134 ± 2 | 173 ± 1 | 173 ± 2 | 0.058 |
| PT-3 | 170 ± 7 | 215 ± 3 | 232 ± 4 | 0.089 |
| PT-4 | 224 ± 3 | 281 ± 7 | 318 ± 11 | 0.138 |

Standard deviation refers to the deviation of the mean value for a triplicate measurement


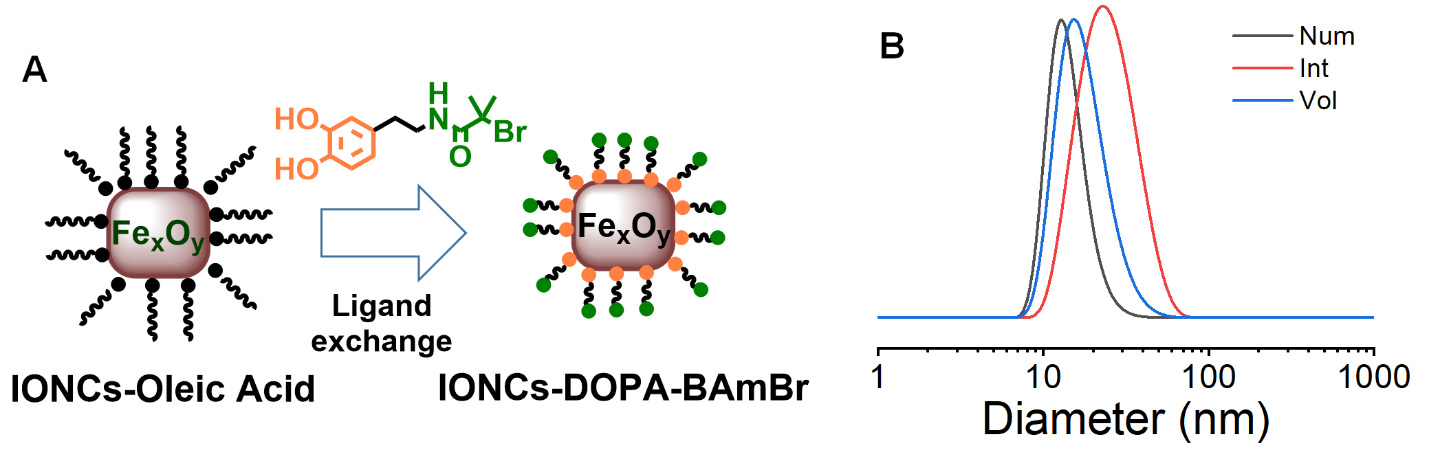


**Figure S2.** (A) The schematic representation of the ligand exchange of IONCs with DOPA-BAmBr to make them soluble in THF/DMF mixture (25%) and (B) DLS traces of IONCs-DOPA-BAmBr in such solvent mixture, a small d_H_ indicates that IONCs are individually dispersed in THF/DMF mixture.


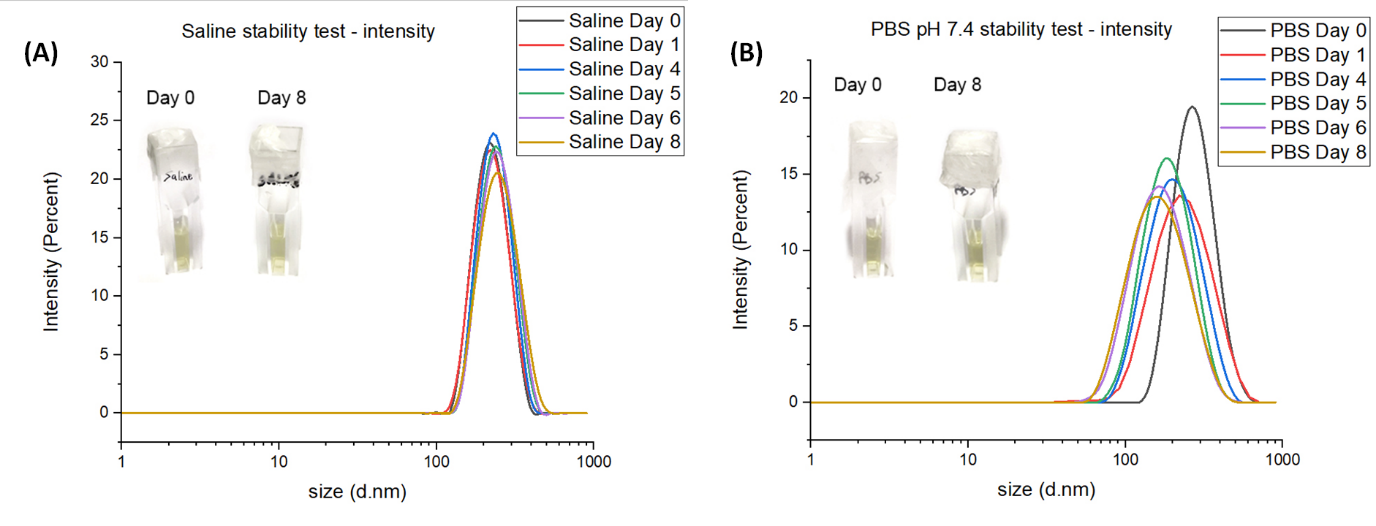


***Figure S3***. *Hydrodynamic Stability of PT-2 photobeads under physiological conditions ( 0.9% NaCl Saline solution) and in PBS buffer, pH 7.4. The DLS traces weighted by intensity were acquired at day 0, 1, 4, 5 6 and 8. Over this period, the samples were stored at ambient conditions in the dark . The insets show images of the cuvettes filled with the photobeads solutions at day 0 and day 8.*


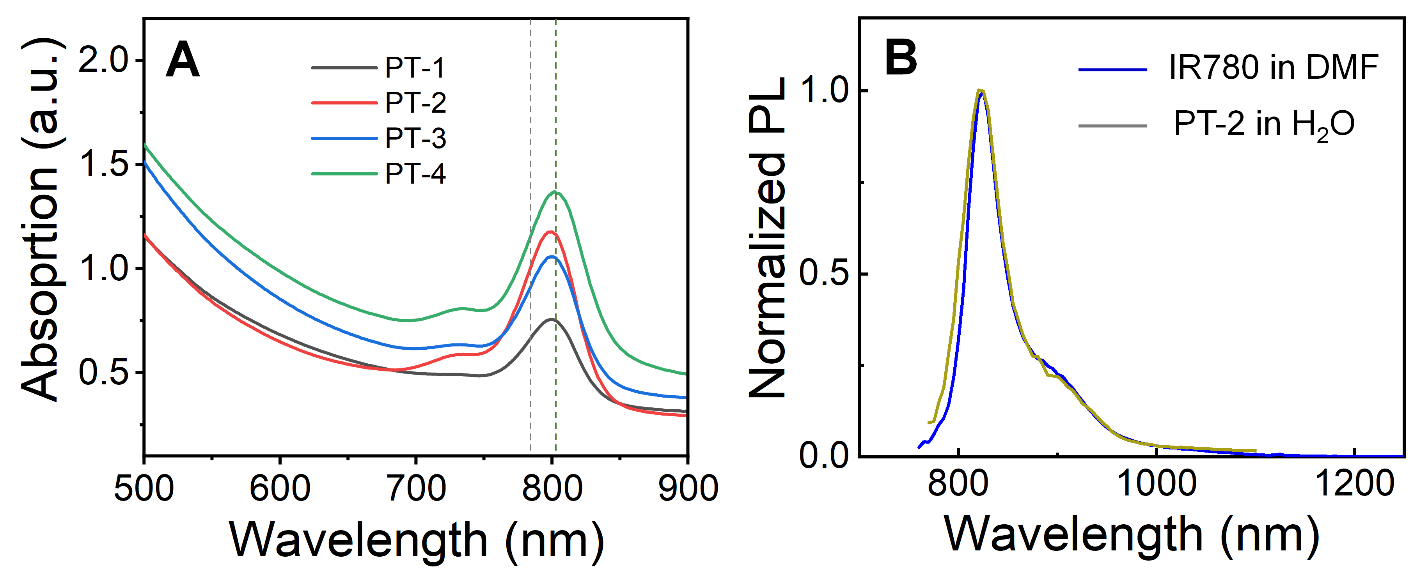


**Figure S4.** (A) Vis-NIR absorption spectra of photobeads containing different amount of IR780. (B) Photoluminescent (PL) spectra of IR780 dye in DMF (blue curve) and photobeads (green curve) that contains 7.2 µg of IR780 per mg of Fe (PT-2).


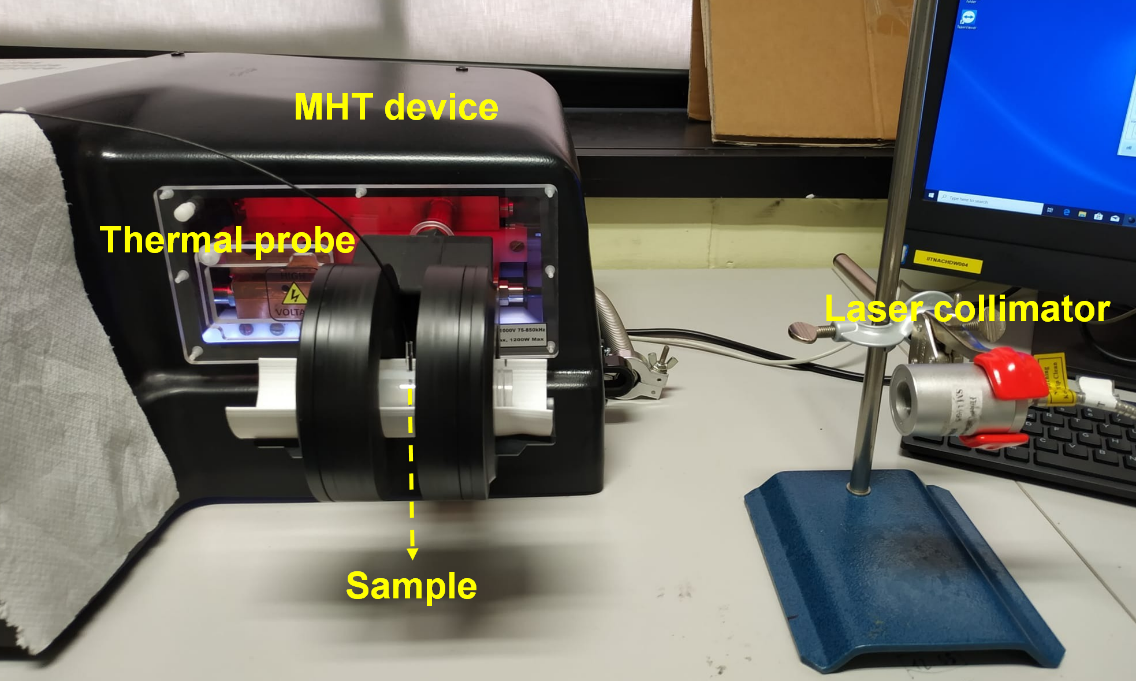


**Figure S5.** Photo of the magnetic hyperthermia and laser set up used in our study for the *in vitro* experiments.


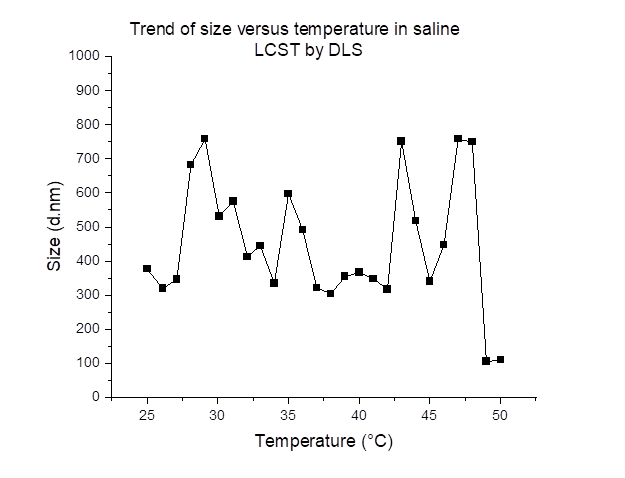

**Figure S6**. Monitoring the DLS size versus temperature for a polymer solution (used to produce the Photobeads) measured in 0.9% saline solution.. In the range 25 to 50°C, that is the range in which we use the beads no marked change in hydrodynamic size was observed indicating a luck of transition temperature of the polymer in this temperature interval.

**MHT coupled with Photothermal treatment measurement – Dye leakage study**

50 µL of photobeads (PT-2, [Fe] = 2.5 g.L*-1*) was loaded in a capillary tube having a diameter of 3 mm and length of 25 mm. The tubes was then placed at the center of the coil of magnetic hyperthermia device using a home-made holder. The temperature probe (optical fiber) was then inserted and adjusted to touch the upper part of solution in order to avoid its direct exposure to laser which will create the interference of temperature readout. Afterwards, the tube was exposed to three cycles of laser irradiation (7 minutes each, wavelength of excitation ( λ ext) 808 nm, 4.67 W/cm*2*) along with 5 minutes of interval. After three laser treatment cycles, the sample was simultaneously exposed to the same laser irradiation and an AMF (24 kA/m and 120 kHz) for another 7 min. For this experiment, the MHT was performed on D5 Series Nanoscale Biomagnetics Corporation. The open coil of this device enables us to apply laser and AMF simultaneously or subsequently as shown in Figure S5. In capillary tubes, both treated (PTT/MHT) and non-treated (control) samples were collected to a magnet (0.3 T ) and after 3 days exposure, the complete separation of the released dye, in solution, from the magnetic photobeads that remained accumulated to the magnet was achieved. The absorption spectra of the supernatant for the control sample and for the treated samples were recorded and compared with each other, as shown in Figure S7.


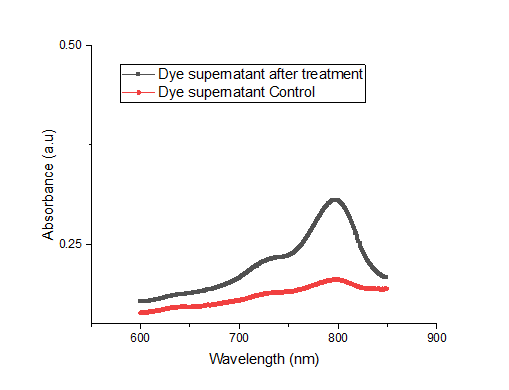


**Figure S7**. Photobeads dye leakage study. UV absorbance spectra of supernatants (red plot – from control sample; black plot – from treated sample (PTT: 4.67 W/cm^2^ ; MHT: f=110 kHz, 19 kA/m).

**Photothermal Conversion Efficiency Study**

To determine the photothermal conversion efficiency of our magnetic photobeads a protocol and the experimental setup (Figure S8) previously reported by some of us was adopted. ^6^ Here, aqueous solution of the photobeads or plain water injected in a glass cuvette contained in a self-made irradiation chamber (Figure S8), were irradiated with laser in vacuum while measuring their heating profiles overtime. After reaching a temperature plateau, the laser was switch off and the cooling curve of the photobead was used to calculate the photothermal conversion efficiency that was determined to be ***ca.* 36 % efficient.**

Photothermal Conversion efficiency Experimental Protocol

In details, prior to irradiation, a 70 × 10^-2^ mbar vacuum was applied to the irradiation chamber by connecting it to a schlenk line. Then 1 mL aqueous solution of photobeads (0.25 mg/mL Fe; Absorbance at 808 nm = 1.28, mass of photobeads (g) = 0.830) was placed into the quartz cuvette (path length = 1 cm). A thermal probe was dipped through the glass tube into the cuvette to touch just the upper part of the photobead solution. Using a laser 808 nm device (laser power 1.13 W, spot size 0.2424 cm^2^), the NPs solution was irradiated for about 15 minutes before being switched off when the solution temperature reached a plateau (ca. 66.4 °C) starting from a temperature of about 19.6°C. The thermal profile of the cooling processed was also recorded using the thermoprobe to yield the representative heating and cooling curve shown in (Figure S8B, red plot). By employing the same set up and laser conditions, the heating-cooling cycle of 1 mL deionized water was also obtained (Figure S8B, black plot).


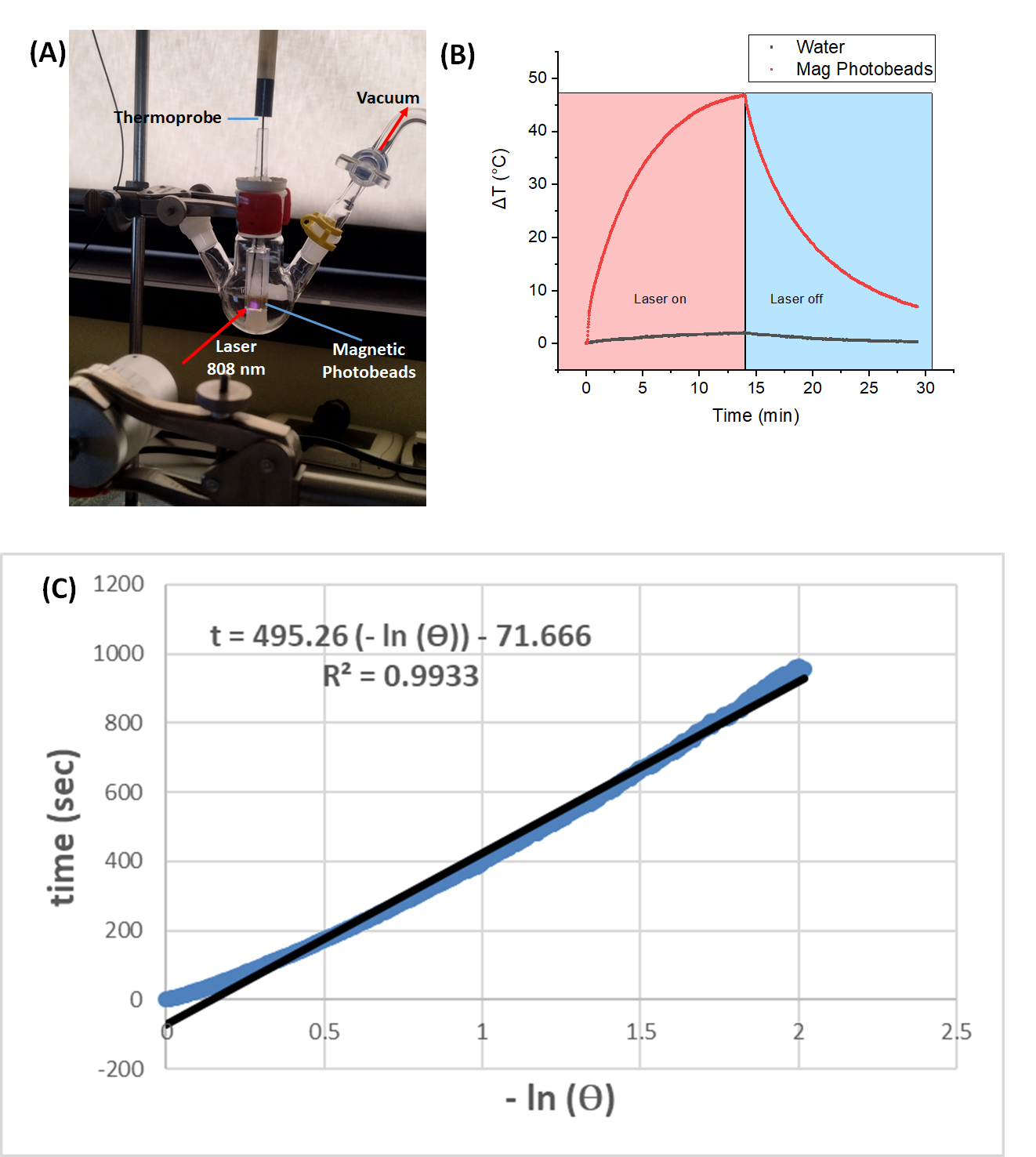


**Figure S8**. (A) The set-up used for the photothermal conversion efficiency determination. A self-made irradiation chamber was designed using a three neck round bottom flask into which a quartz cuvette connected with a glass tube was inserted. Fitted in this chamber also is a thermoprobe to measure the temperature of the NPs solution during laser 808 nm irradiation and (B) Heating-cooling cycle of magnetic photobead solution (red curve) and Milli-Q water (black curve) and (C) Linear fit time/-ln(θ) of the cooling step of magnetic photobead solution.

Using the NP’s cooling curve and employing the total energy balance for the system as expressed by the equation (1), the photothermal conversion efficiency of the photobeads was calculated following a protocol we reported in literatures^6,7^ Details of the equations and data used in the photothermal conversion efficiency calculation are provided here.

The system’s total energy balance is expressed by the equation

$\sum_{\boldsymbol{i}} \boldsymbol{m}_{\boldsymbol{i}}\text{C}\boldsymbol{p}_{\boldsymbol{i}} \frac{\boldsymbol{dT}}{\boldsymbol{dt}}\boldsymbol{=}\boldsymbol{Q}_{\boldsymbol{NC}}\boldsymbol{+}\boldsymbol{Q}_{\boldsymbol{Dis}}\boldsymbol{-}\boldsymbol{Q}_{\boldsymbol{Cond}}$ ( 1)

where *m (g) and Cp* (J·g^-1^·K^-1^) are the mass and specific heat capacity, *T* (K) is the temperature of the solution, *Q_NC_* (W) is the energy input by photobeads under laser irradiation, *Q_Dis_* (W) is the energy input of the water contained in the quartz cuvette and *Q_Cond_* (W) is the heat conduction from the cuvette to the surrounding.

*Q_NC_* is calculated using the equation

$\boldsymbol{Q}_{\boldsymbol{NC}}\boldsymbol{=I}\left( \boldsymbol{1-}\boldsymbol{10}^{\boldsymbol{-A}\boldsymbol{808}} \right)\text{}$ ( 2)

where *I* (W) represents the laser power used during irradiation, A808 is the absorbance of NPs solution at the irradiation wavelength (808 nm) and *h* is the conversion efficiency from incident laser energy to thermal energy.

*Q_Cond_* can be determined by

$\boldsymbol{Q}_{\boldsymbol{Cond}}\boldsymbol{=hS}\left( \boldsymbol{T-}\boldsymbol{T}_{\boldsymbol{amb}} \right)$ ( 3)

where *h* is the heat transfer coefficient (W·cm^-2^·K^-1^), *S* is the surface area (cm^2^) and *T_amb_* the ambient temperature (K). *Q_Cond_* is increasing along with the increase of the temperature during irradiation step and will rise to a maximum when the heat input is equal to the heat output.

$\boldsymbol{Q}_{\boldsymbol{NC}}\boldsymbol{+}\boldsymbol{Q}_{\boldsymbol{Dis}}\boldsymbol{=}\boldsymbol{Q}_{\boldsymbol{Cond-max}}\boldsymbol{=hS(}\boldsymbol{T}_{\boldsymbol{Max}}\boldsymbol{-}\boldsymbol{T}_{\boldsymbol{amb}}\boldsymbol{)}$ ( 4)

The heat efficiency can be obtained by substituting equation (2) in equation (4) obtaining

$\text{}\boldsymbol{=}\frac{\boldsymbol{hS}\left( \boldsymbol{T}_{\boldsymbol{max}}\boldsymbol{-}\boldsymbol{T}_{\boldsymbol{amb}} \right)\boldsymbol{-}\boldsymbol{Q}_{\boldsymbol{Dis}}}{\boldsymbol{I(1-}\boldsymbol{10}^{\boldsymbol{-A}\boldsymbol{808}}\boldsymbol{)}}$ ( 5)

To obtain hS, a dimensionless driving force temperature (ϴ) is introduced using the maximum system temperature (T_max_)

$\boldsymbol{\theta=}\frac{\boldsymbol{T-}\boldsymbol{T}_{\boldsymbol{amb}}}{\boldsymbol{T}_{\boldsymbol{Max}}\boldsymbol{-}\boldsymbol{T}_{\boldsymbol{amb}}}$ ( 6)

and a sample system time constant

$\boldsymbol{\tau}_{\boldsymbol{s}}\boldsymbol{=}\frac{\sum_{\boldsymbol{i}} \boldsymbol{m}_{\boldsymbol{i}}\boldsymbol{C}_{\boldsymbol{p,i}}}{\boldsymbol{hS}}$ ( 7)

Substituting equation (6) and (7) in equation (1) and rearranging, taking into account that during the cooling stage Q_NC_ + Q_dis_ = 0, the equation is reduced to

$\boldsymbol{dt= -}\boldsymbol{\tau}_{\boldsymbol{s}}\frac{\boldsymbol{d\theta}}{\boldsymbol{\theta}}$ ( 8 )

By integrating equation (8) we obtain

$\boldsymbol{t= -}\boldsymbol{\tau}_{\boldsymbol{s}}\boldsymbol{ln(\theta)}$ ( 9 )

From equation (9), the system time constant ($\boldsymbol{\tau}_{\boldsymbol{s}})$ is obtained from the slope of the linear fit of time/-ln(θ) during the cooling step of photobeads solution (see Figure S6C)

By rearranging equation (7) and inserting this t_s_ = 495.26, *hS* was calculated to be 0.018 W K^-1^.

*Q_dis_* was determined from the cooling stage after same laser treatment of the quartz cuvette filled with 1 mL of water. *Q_dis_* was finally calculated following equation (10)

$\boldsymbol{Q}_{\boldsymbol{dis}}\boldsymbol{=hS(}\boldsymbol{T}_{\boldsymbol{MaxWater}}\boldsymbol{-}\boldsymbol{T}_{\boldsymbol{amb}}\boldsymbol{)}$ ( 10 )

resulting in 0.0326 W, since T_max water_-T_amb_ was 1.8 K.

Finally, the photothermal efficiency (h) of the Photobeads was determined from equation (5) using data shown in Table S2:

$\text{}\boldsymbol{=}\frac{\boldsymbol{hS}\left( \boldsymbol{T}_{\boldsymbol{max}}\boldsymbol{-}\boldsymbol{T}_{\boldsymbol{amb}} \right)\boldsymbol{-}\boldsymbol{Q}_{\boldsymbol{Dis}}}{\boldsymbol{I(1-}\boldsymbol{10}^{\boldsymbol{-A}\boldsymbol{808}}\boldsymbol{)}}$ (5)

**Table S2**: Absorbance at irradiation wavelength (Abs_808nm_), mass of photobead solution (m sol), increasing temperature after laser irradiation (ΔT), time system constant (τ_s_), thermal conductance (hS), lase power (I) and photothermal conversion efficiency (Conversion. eff)

| **A808 nm** | **m sol (g)** | **ΔT (°K)** | **τ_s_ (s)** | **h S (W K^-1^)** | **Laser power I (W)** | **Conversion eff. (%)** |
| --- | --- | --- | --- | --- | --- | --- |
| 1.281 | 0.830 | 46.7 | 495.26 | 0.018 | 1.13 | **36.1** |


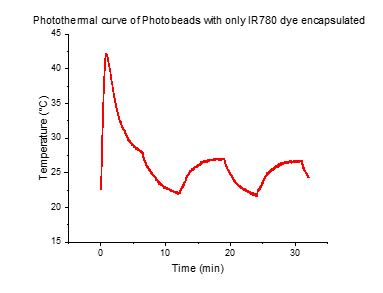


**Figure S9.** Photothermal curve (heating profile) of polymer beads loaded with only IR780 dye and irradiating with a 808 nm laser at 4.67 W/cm^2^ for 3 cycles of 7 minutes each (warming up) followed by 5 min of laser off (cooling curve). This sample containing similar amount of dye of PT-2 formulation. Heat quenching was observed during first cycle of irradiation. This photostability of the dye to irradiation seems different from that of PT-2 sample in which the dye is co-encapsulated with the magnetic nanoparticles.


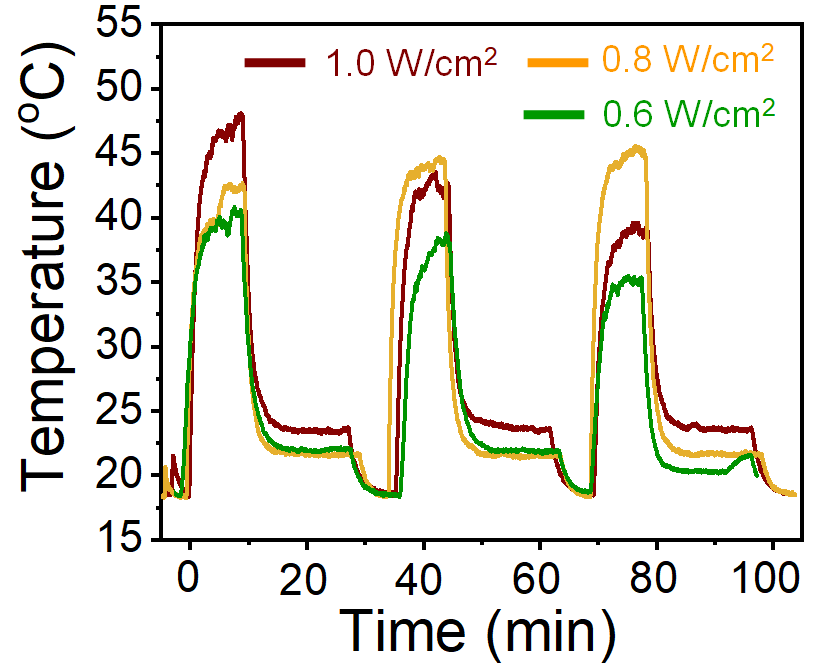


**Figure S10.** Heating profile of the solution containing U87 cell pellets and PT-2 ([Fe] = 3.6 g.L^-1^) upon the simultaneously exposure to MHT (16 kA.m^-1^ and 282 kHz) and laser 808 nm at different power intensities.


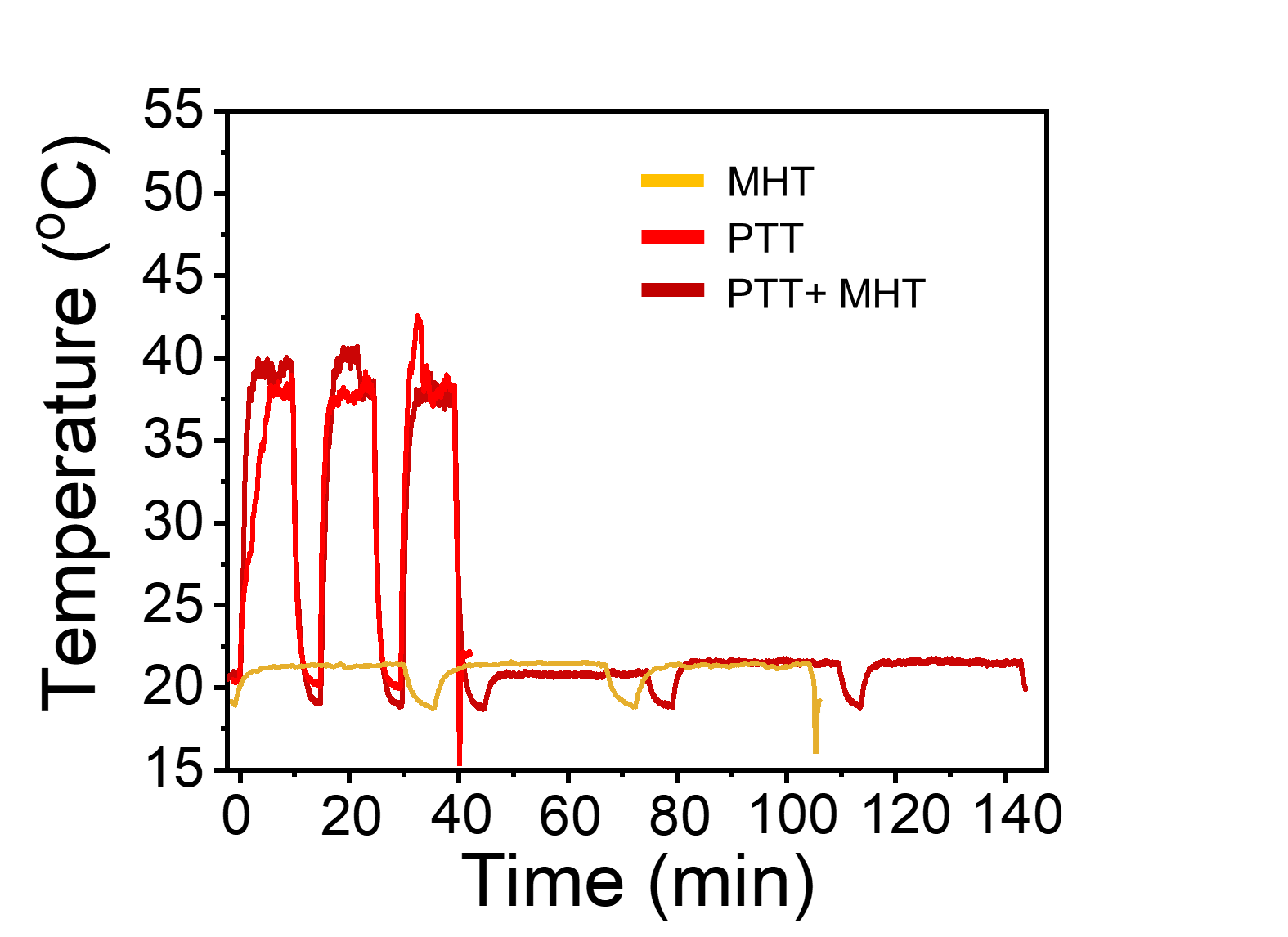


**Figure S11.** Heating profile of the solution containing A431 cell pellets and PT-2 ([Fe] = 3.6 g.L^-1^) upon the subsequently exposure to MHT (16 kA.m^-1^ and 282 kHz) and laser 808 nm (0.7 W/cm^2^). The sample was first exposed to laser and then MHT.


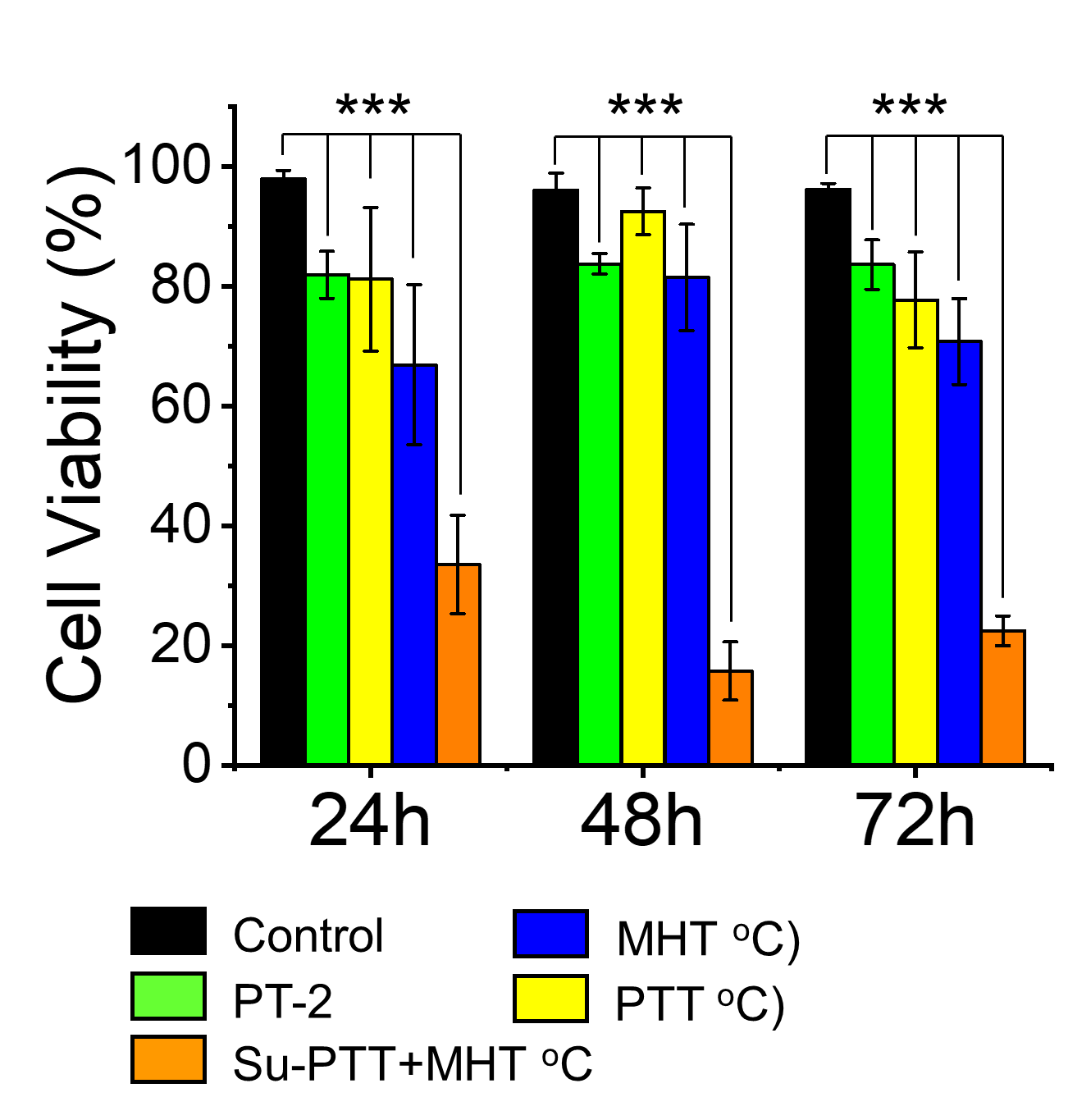


**Figure S12.** Viabilities of A431 epidermoid carcinoma cells that are treated using MHT or PTT or (subsequently) combined PTT and MHT, reaching a maximum therapeutic temperature of 40 ^o^C during PTT or dual PTT and MHT. To note that when MHT was applied the maximum temperature increase corresponded to 23.^o^C. The treatment using MHT and NIR did not show any remarkable toxicity while the combined one resulted in > 80% mortality. Statistical analysis was performed using one-way ANOVA with a Holm-Sidak multiple comparison method. ***p = <0.001.


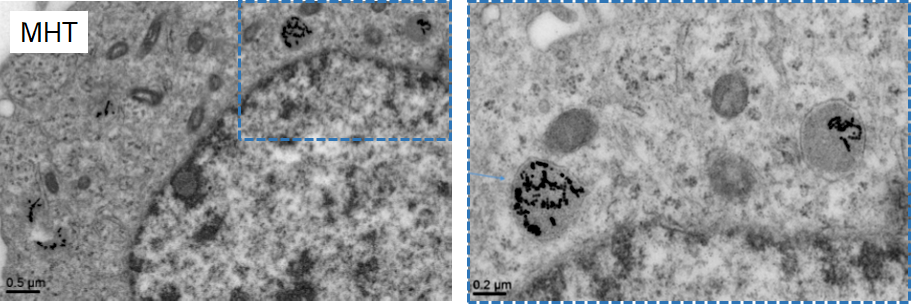


**Figure S13**. TEM images of U87 cells undergone MHT. In this experiment, U87 cell pellets were exposed to PT-2 ([Fe] = 3.6 g.L^-1^) and treated with only MHT (16 kA.m^-1^, 282 kHz, reaching a temperature of 25 ^o^C).


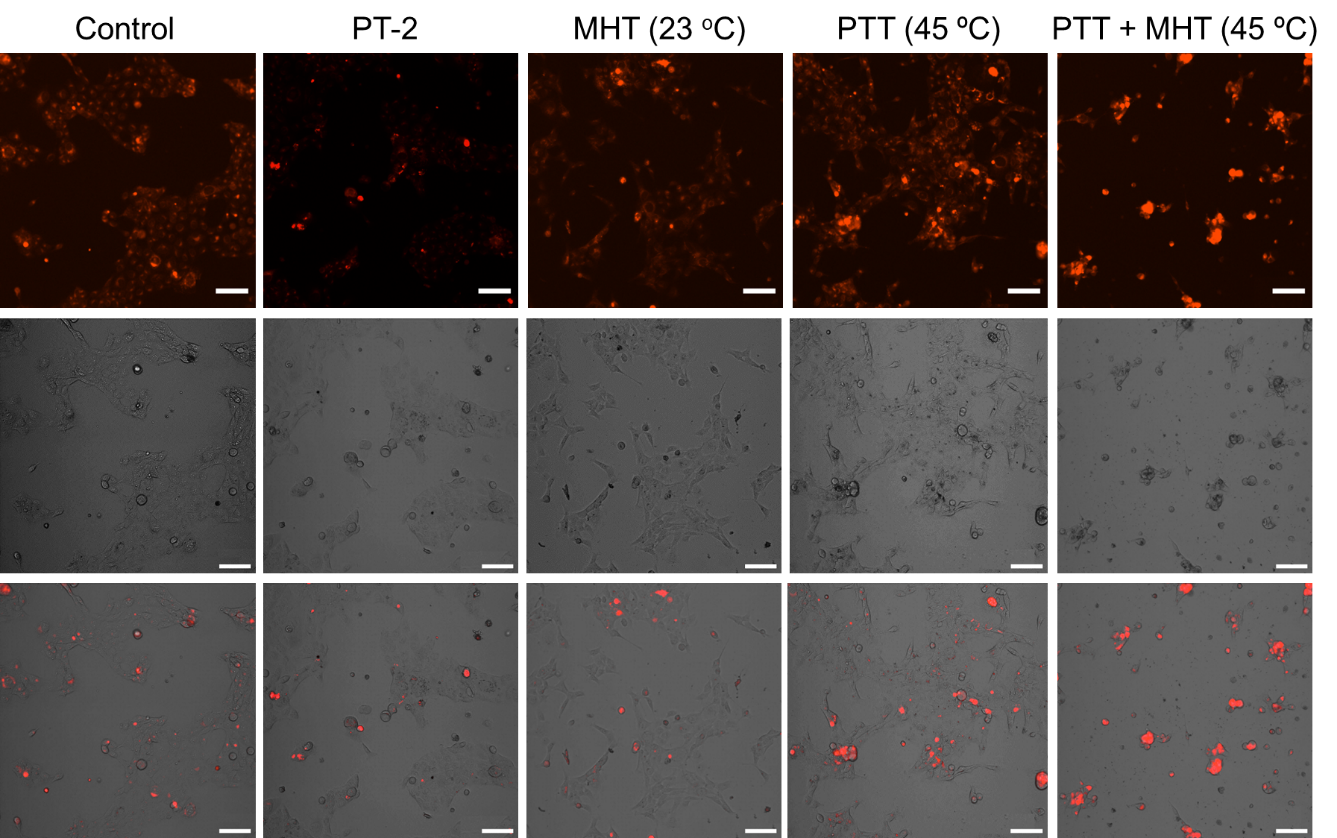


**Figure S14.** Confocal images at 10X magnification of A431 cells upon different treatment conditions stained with Lysotracker fluorophore. In this case, a therapeutic temperature of 40 ^o^C (for standalone PTT and combined MHT and PTT subsequently) was aimed (scale bar: 100 µm). More red fluorescent cells are visible in the sample treated with combined MHT and PTT.


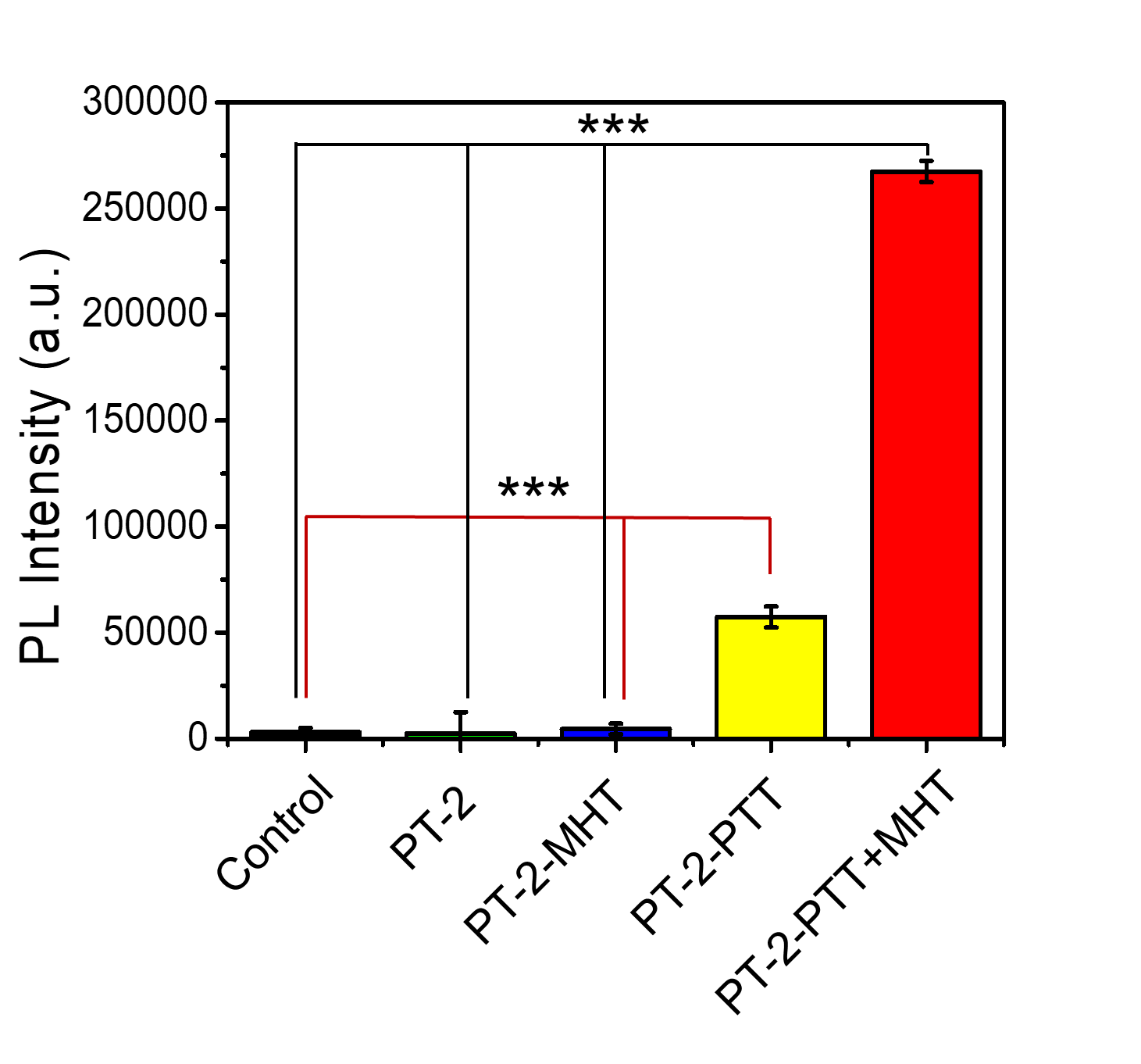


**Figure S15.** Quantitative analysis of fluorescent signal of different A431 cell treated with lysotracker, a pH-sensitive dye after exposure to different treatments. The group treated with subsequent PTT + MHT reaching a maximum temperature at 40 ^o^C showed the highest PL signals, indicating the highest lysosomes activity in this cell group. Statistical analysis was performed using one-way ANOVA with a Dunn´s post hoc test. ***p = <0.001.

1. B. T. Mai, M. J. Barthel, A. Lak, T. Avellini, A. M. Panaite, E. M. Rodrigues, L. Goldoni and T. Pellegrino, *Polymer Chemistry*, 2020, **11**, 2969-2985.

2. B. T. Mai, P. B. Balakrishnan, M. J. Barthel, F. Piccardi, D. Niculaes, F. Marinaro, S. Fernandes, A. Curcio, H. Kakwere and G. Autret, *ACS applied materials & interfaces*, 2019, **11**, 5727-5739.

3. M. V. Zyuzin, M. Cassani, M. J. Barthel, H. Gavilan, N. Silvestri, A. Escudero, A. Scarpellini, F. Lucchesi, F. J. Teran and W. J. Parak, *ACS applied materials & interfaces*, 2019, **11**, 41957-41971.

4. A. Lak, M. Cassani, B. T. Mai, N. Winckelmans, D. Cabrera, E. Sadrollahi, S. Marras, H. Remmer, S. Fiorito and L. Cremades-Jimeno, *Nano letters*, 2018, **18**, 6856-6866.
